# SH2-mediated steric occlusion of the C2 domain regulates autoinhibition of SHIP1 inositol 5-phosphatase

**DOI:** 10.1016/j.jbc.2025.110788

**Published:** 2025-10-07

**Authors:** Emma E. Drew, Hunter G. Nyvall, Matthew A.H. Parson, Reed K. Talus, John E. Burke, Scott D. Hansen

**Affiliations:** 1 Department of Chemistry and Biochemistry, University of Oregon, Eugene, Oregon, USA; 2 Institute of Molecular Biology, University of Oregon, Eugene, Oregon, USA; 3 Department of Biochemistry and Microbiology, University of Victoria, Victoria, British Columbia, Canada; 4 Department of Biochemistry and Molecular Biology, The University of British Columbia, Vancouver, British Columbia, Canada; 5 University of Victoria Genome BC Proteomic Centre, Victoria, British Columbia, Canada

**Keywords:** phosphatase, PI3K, phosphatidylinositol signaling, phosphatidylinositol, phosphatidylinositol phosphatase, Src homology 2 domain, SHIP1

## Abstract

The Src homology 2 (SH2) domain–containing inositol polyphosphate 5-phosphatase 1 (SHIP1) is an immune cell–specific enzyme that regulates phosphatidylinositol-(3,4,5)-trisphosphate signaling at the plasma membrane following receptor activation. SHIP1 plays an important role in processes, such as directed cell migration, endocytosis, and cortical membrane oscillations. Alterations in SHIP1 expression have been shown to perturb myeloid cell chemotaxis and differentiation. In the brain, SHIP1 regulates microglial cell behaviors, which have been linked to Alzheimer’s disease. Understanding the structural and functional relationships of SHIP1 is critical for developing ways to modulate SHIP1 membrane localization and lipid phosphatase activity during immune cell signaling. Recently, we discovered that the N-terminal SH2 domain of SHIP1 suppresses lipid phosphatase activity. SHIP1 autoinhibition can be relieved through interactions with receptor-derived phosphotyrosine peptides presented on membranes or in solution. Using hydrogen–deuterium exchange mass spectrometry, we identified intramolecular contacts between the N-terminal SH2 domain and the CBL1 motif of the C2 domain that limit SHIP1 membrane localization and activity. Single-molecule measurements of purified SHIP1 on supported lipid bilayers and in neutrophil-like cells support a model in which the SH2 domain blocks membrane binding of the central catalytic module. Mutations that disrupt autoinhibition enhance the membrane binding frequency and increase the catalytic efficiency of SHIP1. Although dimerization of SHIP1 enhances membrane localization and the apparent phosphatase activity, it is not required for SHIP1 autoinhibition. Overall, our results provide new insight concerning SHIP1’s structural organization, membrane binding dynamics, and the mechanism of autoinhibition.

Phosphatidylinositol phosphate (PIP) lipids play a critical role in localizing proteins to intracellular membranes in eukaryotic cells (1, 2). The concentration and spatial distribution of PIP lipids are regulated by numerous lipid kinases and phosphatases that compete to establish unique steady-state concentrations required for cellular homeostasis and dynamic membrane signaling responses (3). In the context of cell polarity and migration pathways, receptor activation enhances the plasma membrane localization and activity of PI3K, which drives the rapid phosphorylation of phosphatidylinositol-(4,5)-bisphosphate to generate phosphatidylinositol-(3,4,5)-trisphosphate (PI(3,4,5)P_3_) (4, 5, 6, 7). Functioning downstream of the PI3K signaling pathway, the hematopoietic-specific Src homology 2 (SH2) domain–containing inositol polyphosphate 5-phosphatase 1 (SHIP1) plays a crucial role in regulating the dephosphorylation of PI(3,4,5)P_3_ to produce PI(3,4)P_2_ (8, 9, 10). Genetic KO or silencing of SHIP1 expression perturbs cell polarity and migration in neutrophils (11, 12, 13, 14). SHIP1 KO mice develop chronic myelogenous leukemia characterized by the hyperproliferation and infiltration of myeloid cells in lung tissue (15). In the nervous system, SHIP1 is highly expressed in microglial cells, where it regulates the inflammasome (16, 17). Misregulation of SHIP1 and microglial cell function are markers for Alzheimer’s disease (17, 18). Defining the mechanisms that regulate SHIP1 activation is critical for deciphering how this lipid 5-phosphatase controls myeloid cell signaling.

SHIP1 is a 145 kDa multidomain protein that is predicted to lack secondary structure across ∼30% of its primary amino acid sequence (Fig. 1*A*). The intrinsically disordered C-terminal domain (CTD) (872–1189 amino acids) contains multiple NPxY and proline-rich motifs that can interact with phosphotyrosine (pY) binding and SH3 domain–containing proteins, respectively (19). Positioned in the mostly unstructured peptide sequence connecting the N-terminal SH2 domain and the Pleckstrin homology (PH)-R domain (Fig. 1*A*), the RhoA-binding domain (RBD) has been shown to regulate dimerization of the SHIP1 paralog, SHIP2 (20). Flanking either side of the central 5-phosphatase domain are PH-related and C2 domains (Fig. 1*A*), which display weak affinities for PI(3,4,5)P_3_ and phosphatidylserine (PS) lipids, respectively (21, 22, 23, 24). X-ray crystallography data indicate that the C2 domain forms multiple contacts with the 5-phosphatase domain (21, 23). In the case of SHIP2, the C2 domain modulates the activity of the phosphatase domain, which has led researchers to propose a model of allosteric regulation (23). The residues that bridge the phosphatase and C2 domain interface, however, are not conserved in SHIP1 (21).

**Figure 1:**
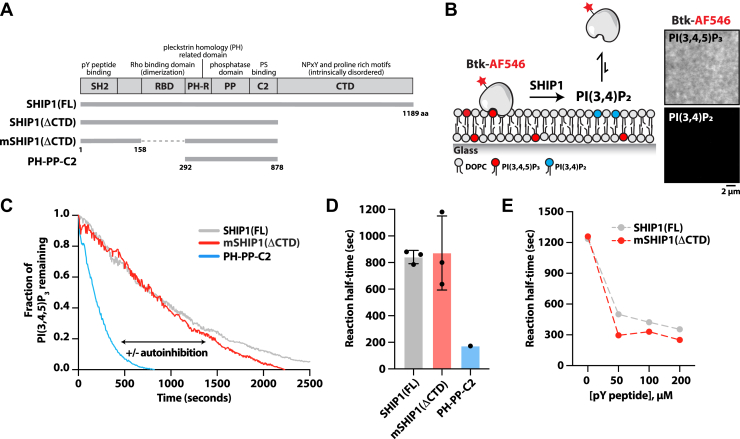
**A minimal SHIP1 protein that exhibits SH2-dependent autoinhibition.***A*, domain organization of SHIP1 proteins biochemically characterized with indicated amino acid numbers. The *dashed line* represents residues in the Rho-binding domain (RBD) that are absent in monomeric SHIP1(ΔCTD). *B*, diagram of the experimental design of *in vitro* SLB experiments to measure SHIP1 activity. Fluorescently labeled Btk1 binds specifically to PI(3,4,5)P_3_ and dissociates from the membrane when SHIP1 dephosphorylates PI(3,4,5)P_3_ to PI(3,4)P_2_. *Inset images* show representative TIRF-M images of the localization of 20 nM Btk-AF546 on 2% PI(3,4,5)P_3_ or 2% PI(3,4)P_2_. *C*, kinetic traces of SHIP1 phosphatase activity in the presence of 20 nM mNG-SHIP1(FL), 20 nM mNG-mSHIP1(ΔCTD), or 20 nM mNG-SHIP1(PH-PP-C2). *D*, quantification of mean reaction half-time measured in the presence of 20 nM mNG-SHIP1(FL), mNG-mSHIP1(ΔCTD), or mNG-SHIP1(PH-PP-C2). N = 3 technical replicates per construct, *p* = 0.868 comparing SHIP1(FL) and mSHIP1(ΔCTD). *E*, tyrosine phosphorylated (pY) peptides stimulate the activation of both mNG-mSHIP1(ΔCTD) and mNG-SHIP1(FL) in solution. For all reactions, dephosphorylation of PI(3,4,5)P_3_ was monitored in the presence of 20 nM Btk-SNAP-AF546. *C*–*E*, initial membrane composition: 2% PI(3,4,5)P_3_, 98% DOPC. Bars represent means. Errors equal SD. *p* Values calculated by the Student's *t* test. Btk1, Bruton’s tyrosine kinase 1; CTD, C-terminal domain; DOPC, 1,2-dioleoyl-sn-glycero-3-phosphocholine; FL, full length; mNG, mNeonGreen; PI(3,4)P2, phosphatidylinositol-(3,4)-bisphosphate; PI(3,4,5)P3, phosphatidylinositol-(3,4,5)-trisphosphate; SH2, Src homology 2; SHIP1, Src homology 2 domain–containing inositol polyphosphate 5-phosphatase 1; SLB, supported lipid bilayer; TIRF-M, total internal reflection fluorescence microscopy.

Previously, we reported that full-length (FL) SHIP1 exhibits autoinhibition based on an undefined set of interactions that depend on the N-terminal SH2 domain (24). Although tyrosine phosphorylated peptides derived from an immunoreceptor tyrosine inhibitory motif (ITIM) can stimulate SHIP1 membrane binding and activity (24), the mechanism of autoinhibition has remained unclear. Here, we present a combination of single-molecule total internal reflection fluorescence microscopy (TIRF-M) and hydrogen–deuterium exchange mass spectrometry (HDX-MS) experiments that elucidates the mechanism of SHIP1 autoinhibition. Solution-based HDX-MS experiments performed in the absence and presence of tyrosine phosphorylated peptides revealed changes in solvent exposure in the N-terminal SH2 and C2 domains, consistent with this being the autoinhibitory interface. Comparing the deuterium exchange rates of monomeric and dimeric SHIP1 revealed similar pY-mediated conformational changes that were limited to the SH2 and C2 domains. Based on the consensus of multiple HDX-MS experiments, we performed mutational analysis to isolate SHIP1 mutants that lack autoinhibition. Similar to the increase in activity observed in the presence of pY peptides, mutations in the CBL1 motif of the C2 domain enhance the membrane binding frequency and increase the catalytic efficiency of SHIP1. Although dimerization enhances SHIP1 membrane localization, we find that a minimal construct lacking the dimerization domain and the unstructured CTD retains the characteristics of the autoinhibited enzyme. Together, our results support a model in which the SH2 domain limits SHIP1 activity by sterically occluding membrane binding of the C2 domain.

## Results

### A minimal SHIP1 protein that exhibits SH2-mediated autoinhibition

Previous biochemical characterization established that SHIP1 autoinhibition is mediated by an undefined set of interactions between the N-terminal SH2 domain and some portion of the central globular domains consisting of the PH-related domain, phosphatase domain, and C2 domain (Fig. 1*A*) (24). Due to the large fraction of intrinsically disordered peptide sequence, purification of FL SHIP1 is challenging and less amenable to structural biochemistry required to decipher the mechanism of autoinhibition. Given the complexity of the FL protein, we aimed to create a minimal SHIP1 construct that exhibits autoinhibition and can be activated with tyrosine phosphorylated (pY) peptides in solution or on supported membranes. Compared with FL SHIP1, our minimal SHIP1 construct lacks the intrinsically disordered CTD. The CTD contributes a minor role to autoinhibition compared with the N-terminal SH2 domain (24). In addition, the minimal SHIP1 construct contains a shorter linker between the SH2 domain and the central domains (Fig. 1*A*). Reducing the linker length removes the RBD, which is predicted to dimerize based on homology to SHIP2 (20). Supporting this model, AlphaFold predicts that the SHIP1(RBD) dimerizes with high confidence (Fig. S1, *A* and *B*). In addition, purified SHIP1(RBD) fused to an mNeonGreen (mNG) eluted from a size-exclusion chromatography column as a single peak with an apparent molecular weight of a dimer (Fig. S1, *C* and *D*). This was observed across multiple protein concentrations, consistent with the isolated RBD forming a constitutive dimer that does not readily dissociate into monomeric species when diluted.

To characterize the phosphatase activity of monomeric SHIP1 (mSHIP1) lacking the CTD (ΔCTD), we used a previously established *in vitro*–supported lipid bilayer (SLB) assay to visualize the dephosphorylation of PI(3,4,5)P_3_ by TIRF-M (24). Serving as a mimetic to the cellular plasma membrane but containing a more simplified lipid composition, these SLBs are fluid and miscible at room temperature. To measure the kinetics of PI(3,4,5)P_3_ dephosphorylation in the presence of recombinantly purified SHIP1, we used a fluorescently labeled lipid-binding domain derived from Bruton’s tyrosine kinase (Btk) as a biosensor (Fig. 1*B*). Compared with the Grp1-derived PI(3,4,5)P_3_ sensor used in our previous study (24), here we used a monomeric Btk mutant protein that interacts with a single PI(3,4,5)P_3_ lipid. This biosensor exhibits more rapid equilibration kinetics and a faster dissociation rate constant compared with Grp1 (25, 26). Using this assay, we measured the lipid phosphatase activity of FL SHIP1 and mSHIP1(ΔCTD) (Fig. 1, *C* and *D*). Both proteins exhibited ∼5-fold reduction in phosphatase activity compared with the central catalytic module of SHIP1 alone, which we refer to as PH-PP-C2 (Fig. 1, *C* and *D*). The difference in activity between mSHIP1(ΔCTD) and constitutively active SHIP1(PH-PP-C2) construct sets the dynamic range for characterizing SHIP1 mutants that modulate lipid phosphatase activity. The addition of a pY peptide derived from an ITIM of FCγRIIB in solution relieved autoinhibition and stimulated the phosphatase activity to similar extents for both FL SHIP1 and mSHIP1(ΔCTD) (Fig. 1*E*).

### HDX-MS reveals pY peptide–mediated conformational changes in autoinhibited SHIP1

**Figure 2:**
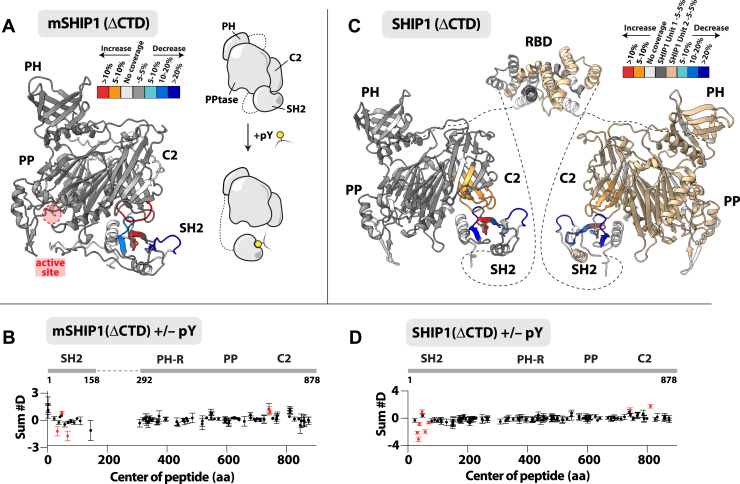
**HDX-MS reveals intramolecular contacts between the SH2 and C2 domain of SHIP1.***A*, peptides in the SH2 and C2 domains of monomeric SHIP1(ΔCTD) (1.5 μM) showing significant changes in HDX (>0.4 Da and 5% difference, with a two-tailed *t* test *p* < 0.01) when incubated with 40 μM pY peptide are mapped onto an AlphaFold2 model for monomeric mSHIP1(ΔCTD) (also refer to Fig. S3). Differences in exchange are color coded according to the legend. Cartoon model summarizing pY-mediated conformational changes in mSHIP1(ΔCTD) is shown to the *right*. *B*, sum of the number of deuteron differences for mSHIP1(ΔCTD) measured in the absence or presence of 40 μM pY peptide. *C*, autoinhibited SHIP1(ΔCTD) (1.5 μM) showing significant changes in HDX (>0.4 Da and 5% difference with a two-tailed *t* test *p* < 0.01) when incubated with 40 μM pY peptide are mapped onto an AlphaFold3 model for dimeric SHIP1. Dimerization of the Rho-binding domain (RBD) was inferred from AlphaFold3 structure prediction. Generation of dimer model from AlphaFold3 output is shown in Fig. S4. *Dashed lines* represent linkers that are predicted to lack secondary structure. Differences in exchange are color coded according to the legend. *D*, sum of the number of deuteron differences for SHIP1(ΔCTD) measured in the absence or presence of 40 μM pY peptide. *B* and *D*, values are based on the integrated difference in exchange over the entire time course. Each point represents a single peptide, and error bars are shown as the sum of the standard deviation across all time points (n = 3 for each time point). Peptides with a statistically significant difference in HDX are highlighted in *red*, with the domain schematic annotated above. See Fig. S2 for a more complete set of peptides comparing apo mSHIP1(ΔCTD) and SHIP1(ΔCTD) deuterium incorporation in the absence and presence of pY peptide. Refer to *Experimental procedures* section and Tables S1 and S2 for full description of statistical evaluation method. CTD, C-terminal domain; HDX-MS, hydrogen–deuterium exchange mass spectrometry; pY, phosphotyrosine; SH2, Src homology 2; SHIP1, Src homology 2 domain–containing inositol polyphosphate 5-phosphatase 1.

The structure of FL autoinhibited SHIP1 has not been solved by X-ray crystallography or cryo-electron microscopy. To determine the structural basis of SHIP1 autoinhibition, we performed HDX-MS experiments to identify peptide sequences that regulate intramolecular interactions between the N-terminal SH2 domain and presumably the central catalytic core flanked by the PH and C2 domains. HDX-MS measures the exchange rate of amide hydrogens acting as a readout of protein secondary structure dynamics. HDX-MS is capable of identifying both direct and allosteric changes, which occur in the presence of ligand (27). We performed solution experiments with mSHIP1(ΔCTD) in the absence and presence of pY-ITIM peptides (Fig. 2*A*). The HDX-MS experiments identified SHIP1 peptides in the SH2 domain that exhibited both increased exchange (46–52 amino acids) and decreased exchange (29–39 and 64–72 amino acids), as well as peptides in the C2 domain that displayed an increase in exchange in the presence of the pY-ITIM peptide (Figs. 2, *A* and *B* and S2*A*). In particular, the disordered CBL1 motif in the C2 domain (740–748 amino acids) displayed increased solvent exposure in the presence of pY-ITIM peptides. In agreement with our HDX-MS data, AlphaFold (28, 29) consistently predicted an autoinhibited mSHIP1(ΔCTD) structure with potential intramolecular contacts between the SH2 and C2 domains (Figs. 2*A* and S3, *A* and *B*). The predicted structure for the SHIP1(PPtase-C2) was in good agreement with the SHIP1 and SHIP2 (PPtase-C2) structures previously solved by X-ray crystallography (21, 23) (Fig. S3*C*).

To determine whether the SHIP1 dimerization motif modulates pY peptide–mediated solvent exposure of the SH2 and C2 domains, we performed HDX-MS experiments using SHIP1(ΔCTD) (Fig. 2*C*). This construct lacks the disordered CTD but contains the RBD dimerization domain (152–291 amino acids). Using AlphaFold3 (30), we mapped pY peptide–dependent changes in HDX to a model of SHIP1(ΔCTD) in a dimeric state (Figs. 2, *C* and *D* and S4). Overall, the HDX-MS experiments performed with SHIP1(ΔCTD) identified intramolecular contacts between the SH2 and the CBL1 motif in agreement with our results using the mSHIP1(ΔCTD) construct lacking the RBD (Fig. 2*A*). In addition to observing solvent exposure of the CBL1 motif, the HDX-MS results also revealed a second loop exposure in the CBL3 motif of the C2 domain (Fig. 2, *C* and *D*). Note that the AlphaFold3 structure prediction tries to maximize the surface area of intersubunit contact in the SHIP1 dimer (Fig. S4*A*), particularly in the C2 and SH2 domains. However, the predicted alignment error for dimer interfaces outside the RBD was predicted with very low confidence, with a high confidence predicted alignment error prediction for the RBD dimer (Fig. S4*B*). In addition, there is no structural biochemistry to support the proposed AlphaFold3 intramolecular interactions beyond those mediated by the dimerization domain (Fig. S4, *B*–*D*). As a result, our structural model assumes that the RBD is the only point of contact between two SHIP1 subunits in a dimer (Figs. 2*C* and S4*D*).

### Mutations in the C2 domain disrupt SHIP1 autoinhibition

**Figure 3:**
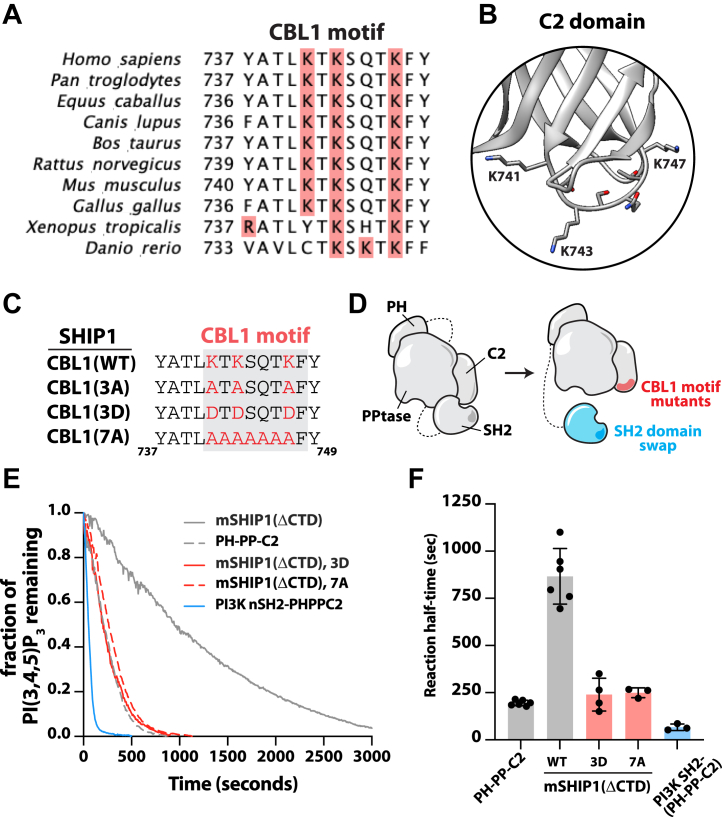
**Mutations in the SH2 domain or CBL1 motif disrupt SHIP1 autoinhibition.***A*, sequence alignment of CBL1 motif across vertebrate species. Highlighted in *red* are positively charged residues within the CBL1 motif. *B*, zoom in structural model of human SHIP1 CBL1 motif with indicated lysine residues (Protein Data Bank code: 6IBD). *C*, sequence alignment summarizing human SHIP1 CBL1 motif mutant generated in this study. *D*, cartoons of SHIP1 autoinhibition mutants. *E*, SHIP1 CBL1 motif mutants and SH2 domain swap display increased phosphatase activity. Kinetic traces of phosphatase activity measured in the presence of 20 nM mNG-mSHIP1(ΔCTD) (WT and mutants). *F*, quantification of reaction half-times of 20 nM mNG-SHIP1(PH-PP-C2), mNG-mSHIP1(ΔCTD), and mNG-mSHIP1(ΔCTD) mutants (N = 3–6 technical replicate per construct). Statistical significance evaluated by the Student's *t* test comparing mSHIP1(ΔCTD) to the following mutants: 3D (∗*p* < 0.0001), 7A (∗*p* < 0.0001), PI3K(SH2)-SHIP1(PH-PP-C2) (∗*p* < 0.0001). Bars represent mean. Errors equal SD. *p* Values calculated by the Student's *t* test. *E* and *F*, initial membrane composition: 2% PI(3,4,5)P_3_, 98% DOPC. For all SHIP1 phosphatase activity assays, dephosphorylation of PI(3,4,5)P_3_ was monitored in the presence of 20 nM Btk-SNAP-AF546. Btk, Bruton’s tyrosine kinase; CTD, C-terminal domain; DOPC, 1,2-dioleoyl-*sn*-glycero-3-phosphocholine; SH2, Src homology 2; SHIP1, Src homology 2 domain–containing inositol polyphosphate 5-phosphatase 1; mNG, mNeonGreen; PI(3,4,5)P_3_, phosphatidylinositol-(3,4,5)-trisphosphate.

Based on our HDX-MS data, we hypothesized that the SH2 domain binds to the C2 domain, which limits SHIP1 interactions with membrane lipids such as PS. Intramolecular interactions between the SH2 and C2 domains could also prevent the ability of the C2 domain to change conformation, which has been proposed to allosterically regulate 5-phosphatase activity of SHIP2 (23). To determine the functional significance of the SH2–C2 intramolecular interactions, we purified mSHIP1(ΔCTD) containing mutations in the conserved CBL1 motif (Fig. 3*A*). The SHIP1 CBL1 motif contains multiple positively charged lysine residues that could mediate electrostatic interdomain interactions (Fig. 3, *A* and *B*). We purified mSHIP1(ΔCTD) mutants with the lysine residues mutated to uncharged alanine residues (K741A/K743A/K747A; denoted 3A) or negatively charged aspartic acid residues (K741D/K743D/K747D; denoted 3D) (Fig. 3*C*). In addition, we mutated all the residues in the disordered CBL1 motif to alanine residues (denoted 7A) (Fig. 3, *C* and *D*). Using our SLB TIRF-M assay, we measured the phosphatase activity of the SHIP1 CBL1 mutants compared with autoinhibited mSHIP1(ΔCTD) and the constitutively active central domain (*i.e*., PH-PP-C2) (Fig. 3*E*). Mutating three CBL1 motif residues to uncharged residues (*i.e*., 3K to 3A) did not alter the phosphatase activity of mSHIP1(ΔCTD) (Fig. S5, *A*–*C*). In contrast, the mSHIP1(ΔCTD) containing the 3D charge swap or 7A loop mutant displayed increased 5-phosphatase activity with kinetic traces indistinguishable from constitutively active SHIP1(PH-PP-C2) (Fig. 3, *E* and *F*).

The AlphaFold prediction of autoinhibited mSHIP1(ΔCTD) did not reveal an obvious patch of negatively charged residues on the SH2 domain that could facilitate interactions with the CBL1 motif of the C2 domain (Figs. 2*C* and S4). Interpreting the HDX-MS data is also challenging because pY peptide binding can mask SH2 domain solvent exposure when SHIP1 autoinhibition is relieved. SH2 domain residues shielded by the pY peptide could also be responsible for both SHIP1 autoinhibition and pY peptide binding. To determine the role of the SH2 domain in regulating autoinhibition, we instead made a chimeric protein containing the nSH2 domain derived from human PI3Kα fused to SHIP1(PH-PP-C2) in the same molecular configuration of mSHIP1(ΔCTD) (Fig. 3*D*). Compared with our minimal construct, mSHIP1(ΔCTD), the PI3Kα(nSH2)-PH-PP-C2 chimera displayed enhanced 5-phosphatase activity (Fig. 3, *E* and *F*). Interestingly, the catalytic activity of the chimera was slightly greater than the 3D and 7A mutants that disrupt SHIP1 autoinhibition. Based on our single-molecule TIRF-M measurements, the increase in activity can be explained by a 2.6-fold increase in membrane binding frequency for PI3Kα(nSH2)-PH-PP-C2 compared with the SHIP1(PH-PP-C2) construct (Fig. S6, *A* and *B*).

### CBL1 motif mutants maintain sensitivity to PS lipids

**Figure 4:**
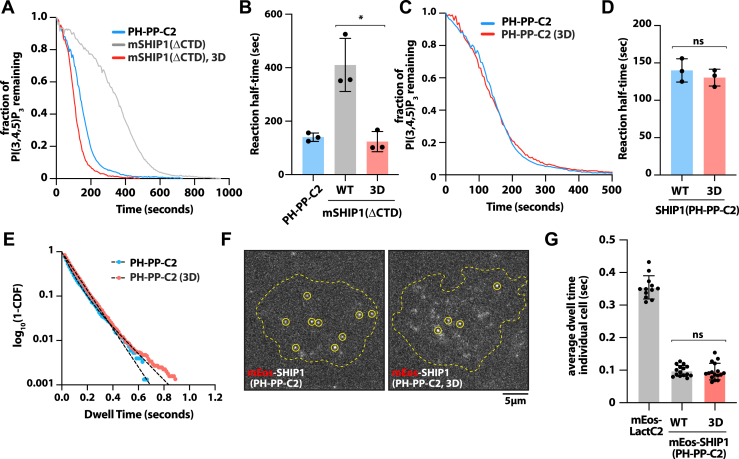
**Phosphatidylserine lipids similarly enhance phosphatase activity of SHIP1 and CBL1 motif mutants.***A*, lipid phosphatase activity measured in the presence of 2 nM mNG-SHIP1(PH-PP-C2) and mNG-mSHIP1(ΔCTD) (WT and mutants). *B*, quantification of reaction half-times of PH-PP-C2, mSHIP1(ΔCTD), and mSHIP1(ΔCTD) (K741D/K743D/K747D; denoted 3D). Bars equal mean reaction half-times. (N = 3 technical replicates per construct). *p* = 0.009 comparing mSHIP1(ΔCTD) and mSHIP1(ΔCTD, 3D). *C*, phosphatase activity measurements of 2 nM mNG-SHIP1(PH-PP-C2) WT and 3D mutant. *D*, quantification of reaction half-time of data in (*C*). (N = 3 technical replicates per construct, *p* = 0.432, ns). *E*, single-molecule dwell time distributions for all mNG-SHIP1 membrane–binding events. Dwell times were calculated by fitting log_10_(1-cumulative distribution frequency [CDF]) to a single exponential decay curve (*black dashed lines*). *F*, representative TIRF-M images showing single-molecule plasma membrane localization of indicated mEos-tagged SHIP1 construct in PLB-985 cells. *G*, average single-molecule membrane dwell times for mEos-SHIP1(PH-PP-C2) (N = 15 cells), mEos-SHIP1(PH-PP-C2, 3D) (N = 16 cells), and mEos-LactC2 (N = 12 cells). Each data point is the average dwell measured for an individual cell. *p* = 0.806 comparing mEos-SHIP1(PH-PP-C2) and mEos-SHIP1(PH-PP-C2, 3D). *A*–*E*, membrane composition: 2% PI(3,4,5)P_3_, 20% DOPS, and 78% DOPC. For all kinetic assays, dephosphorylation of PI(3,4,5)P_3_ was monitored in the presence of 20 nM Btk-SNAP-AF546. Errors equal SD. *p* Values were calculated by the Student’s *t* test. Btk, Bruton’s tyrosine kinase; CTD, C-terminal domain; DOPC, 1,2-dioleoyl-*sn*-glycero-3-phosphocholine; DOPS, 1,2-dioleoyl-*sn-*glycero-3-phospho-l-serine; mNG, mNeonGreen; PI(3,4,5)P_3_, phosphatidylinositol-(3,4,5)-trisphosphate; SHIP1, Src homology 2 domain–containing inositol polyphosphate 5-phosphatase 1; TIRF-M, total internal reflection fluorescence microscopy.

Previous work established that PS lipids enhance SHIP1/2 phosphatase activity independent of autoinhibition (23, 24). Since C2 domains are known for binding PS lipids (31), we wanted to determine the functional significance of the CBL1 motif in controlling PS-mediated enhancement of SHIP1 phosphatase activity. Using the SLB TIRF assay to monitor phosphatase activity, we created bilayers with a physiologically relevant concentration of 20% PS lipids to measure the activity of the CBL1 mutants (32). The activity of mSHIP1(ΔCTD), mSHIP1(ΔCTD, 3D), and SHIP1(PH-PP-C2) followed the same trend as activity measurements performed on bilayers in the absence of PS lipids (Fig. 4, *A* and *B*). However, these experiments used a 10-fold lower concentration of SHIP1 protein to produce similar reaction half-times measured in the absence of PS lipids. In agreement with previous research, the enhanced activity was independent of autoinhibition since constructs lacking the SH2 domain also exhibit increased catalytic efficiency.

Independent of intramolecular interactions between the SH2 and C2 domains, mutations in the CBL1 motif could stimulate 5-phosphatase activity through an allosteric mechanism based on altered interdomain communication between the central phosphatase and C2 domains. This would be surprising given that the CBL1 motif is positioned distal to residues that form the interface between the phosphatase and C2 domains to regulate allostery (21, 22, 23). To test whether CBL1 motif mutations modulate phosphatase activity independent of the proposed autoinhibition mechanism, we introduced the CBL1 motif mutations (*i.e*., 3K to 3D) into the constitutively active SHIP1 construct (*i.e*., PH-PP-C2). When we measured the activity of SHIP1(PH-PP-C2) and SHIP1(PH-PP-C2, 3D) in the presence of 2% PI(3,4,5)P_3_ and 20% PS lipids, we observed nearly identical kinetic traces and reaction half-times (Fig. 4, *C* and *D*). Consistent with these data, single-molecule dwell time analysis comparing mNG-SHIP1(PH-PP-C2), WT, and 3D mutant yielded dwell times that were indistinguishable (Fig. 4*E* and Table 1). Lateral diffusion of membrane-bound mNG-SHIP1(PH-PP-C2), WT, and 3D mutant was also nearly identical (Fig. S7 and Table 1). This indicates that the charge swap mutations in the CBL1 motif do not affect the frictional drag coefficient or membrane contact surface area of the mNG-SHIP1(PH-PP-C2) protein. Overall, mutations in the CBL1 motif do not impact PS lipid binding of SHIP1 *in vitro*. When we measured SHIP1 activity on SLBs lacking PS lipids, however, we observed a 22% increase in the activity of the CBL1 mutant compared with the WT phosphatase (Fig. S5, *D* and *E*). This can be rationalized by the lack of 20% PS lipids in the SLBs, which reduces charge repulsion and causes a small amount of nonspecific binding of the 3D CBL1 mutant. Importantly, the magnitude of this difference cannot explain the ∼3.5-fold enhancement in activity observed when the 3D or 7A CBL1 mutants were introduced into the autoinhibited mSHIP1(ΔCTD) construct (Fig. 3*F*).

**Table 1:** Single-molecule analysis of mNG-SHIP1 on SLBs

Protein visualized	τ_1_ ± SD (s)	τ_2_ ± SD (s)	α ± SD	*N*	*n*
mNG-mSHIP1 (ΔCTD)	0.409 ± 0.009	—	—	3	12,813
mNG-SHIP1 (ΔCTD)	0.359 ± 0.007	1.83 ± 0.03	0.74 ± 0.002	2	8451
mNG-SHIP1 (ΔCTD), dimers	1.79 ± 0.001	—	—	2	939
mNG-SHIP1 (FL)	0.333 ± 0.03	1.81 ± 0.08	0.49 ± 0.02	3	7888
mNG-SHIP1 (FL), dimers	2.11 ± 0.14	—	—	3	1680
mNG-SHIP1 (PH-PP-C2)	0.083 ± 0.011	—	—	4	13,495
mNG-SHIP1 (PH-PP-C2), 3D	0.078 ± 0.011	—	—	4	18,784


Protein visualized	D1 ± SD (μm^2^/s)	D2 ± SD (μm^2^/s)	α ± SD	*N*	Steps
mNG-SHIP1 (PH-PP-C2)	0.196 ± 0.015	1.61 ± 0.243	0.79 ± 0.03	4	110,095
mNG-SHIP1 (PH-PP-C2), 3D	0.208 ± 0.025	1.98 ± 0.431	0.76 ± 0.02	4	139,805
mNG-mSHIP1 (ΔCTD)	0.065 ± 0.005	0.429 ± 0.004	0.31 ± 0.007	3	117,808
mNG-SHIP (ΔCTD)	0.035 ± 0.003	0.226 ± 0.017	0.42 ± 0.009	2	125,409
mNG-SHIP1 (FL)	0.028 ± 0.001	0.164 ± 0.003	0.34 ± 0.017	3	168,117

SD is from the indicated number of technical replicates; *N* = # of SLBs for calculating the mean dwell times (*i.e*., technical replicates); *n* = total number of molecules tracked across the indicated number of technical replicates (*N*); alpha (α) = fraction of molecules with characteristic dwell time (τ_1_) or diffusion coefficient (D1). The fraction of long dwelling species (τ_1_) or fast diffusing molecules (D1) equals 1–α. Steps = total number of displacements measured for all single-molecule trajectories from all technical replicates (*N*). Membrane composition for mNG-mSHIP1(DCTD) and mNG-SHIP1(ΔCTD and FL): 2% PI(3,4,5)P_3_, 2% MCC-PE, 96% DOPC with pY-conjugated peptide. Membrane composition for mNG-SHIP1(PH-PP-C2, WT, and 3D): 2% PI(3,4,5)P_3_, 20% DOPS, and 78% DOPC. Photobleaching kinetics of mNG-SHIP1(PH-PP-C2) immobilized on glass yielded a time constant (τ_bleach_) of 2.7 s.

To measure SHIP1 membrane binding properties on a more complex membrane, we expressed SHIP1(PH-PP-C2) and SHIP1(PH-PP-C2, 3D) mutants as mEos3.2 fusion proteins in PLB-985 neutrophil–like cells as previously described (24). Stable lentiviral-based expression resulted in a low level of expression with uniform localization across the plasma membrane. To resolve single membrane–bound molecules, a fraction of mEos-SHIP1 proteins were photoconverted from the green to red fluorescent state through transient exposure to 405 nm light (Fig. 4*F*). This allowed us to visualize a single mEos-SHIP1 molecule and measure its membrane binding properties *in vivo* (Movie S1). Comparing mEos-SHIP1(PH-PP-C2) and mEos-SHIP1(PH-PP-C2, 3D) across numerous cells revealed no significant difference in the average membrane dwell time (Fig. 4*G*). Consistent with our dwell time analysis of mNG-SHIP1(PH-PP-C2) on SLBs, dwell times measured on the plasma membrane of immune cells were nearly identical to our *in vitro* measurements (Table 1). To determine if our ability to measure differences between mEos-SHIP1 constructs was masked by a photobleaching artifact, we quantified the dwell time of plasma membrane–localized mEos-LactC2. This lipid-binding domain interacts strongly with PS and exhibits an average membrane dwell time ∼3.5 times greater than our mEos-SHIP1 constructs (Fig. 4*G*).

### Autoinhibition reduces the membrane binding frequency of SHIP1

**Figure 5:**
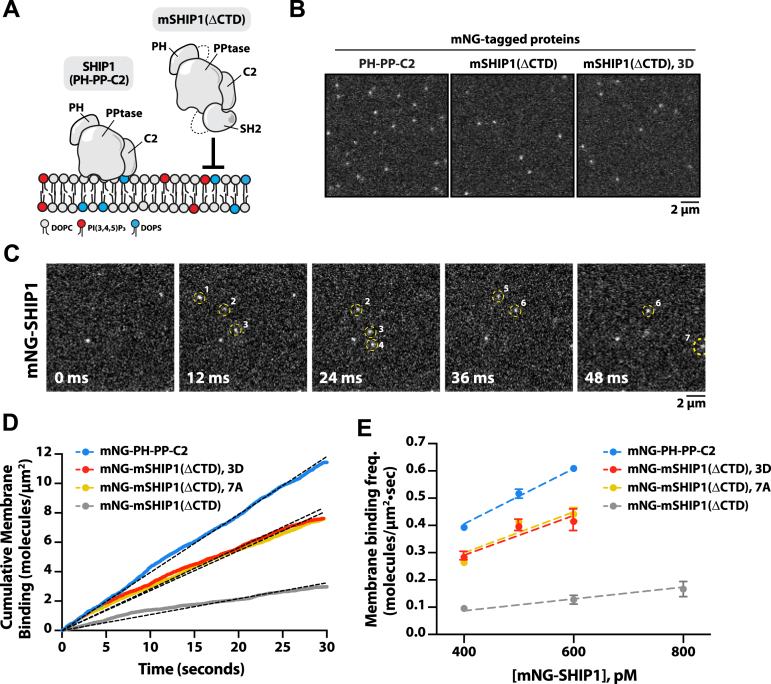
**SH2-mediated steric occlusion of the CBL1 motif reduces the membrane binding frequency of SHIP1.***A*, cartoon illustrating predicted difference in membrane binding for SHIP1(PH-PP-C2) and mSHIP1(ΔCTD) based on SH2-mediated steric occlusion of the C2 domain. *B*, representative TIRF-M images of membrane localization of 400 pM mNG-SHIP1(PH-PP-C2), 400 pM mNG-mSHIP1(ΔCTD), and 400 pM mNG-mSHIP1(ΔCTD, K741D/K743D/K747D; denoted 3D). *C*, representative time sequence of images showing the frame-by-frame localization of mNG-SHIP1. New membrane binding events are scored and cumulated to calculate the binding frequency. *D*, cumulative binding events measured in the presence of 400 pM mNG-SHIP1(PH-PP-C2), mNG-mSHIP1(ΔCTD), mNG-mSHIP1(ΔCTD, 3D), or mNG-mSHIP1(ΔCTD, 7A). *E*, membrane binding frequency (*k*_ON_) calculated from measuring cumulative binding events across the following protein concentrations: 400 to 600 pM mNG-SHIP1(PH-PP-C2), 400 to 600 pM mNG-mSHIP1(ΔCTD, 3D), 400 to 600 pM mNG-mSHIP1(ΔCTD, 7A), and 400 to 800 pM mNG-mSHIP1(ΔCTD). Linear regression yielded the following *k*_ON_ values: 1.0 nM^−1^ μm^−2^•s^−1^ mNG-SHIP1(PH-PP-C2), 0.73 nM^−1^ μm^−2^•s^−1^ mNG-mSHIP1(ΔCTD, 3D), 0.75 nM^−1^ μm^−2^•s^−1^ mNG-mSHIP1(ΔCTD, 7A), and 0.22 nM^−1^ μm^−2^•s^−1^ mNG-mSHIP1(ΔCTD). *B*–*E*, membrane composition: 2% PI(3,4,5)P_3_, 20% DOPS, and 78% DOPC. CTD, C-terminal domain; DOPC, 1,2-dioleoyl-*sn*-glycero-3-phosphocholine; DOPS, 1,2-dioleoyl-*sn-*glycero-3-phospho-l-serine; mNG, mNeonGreen; PI(3,4,5)P_3_, phosphatidylinositol-(3,4,5)-trisphosphate; SH2, Src homology 2; SHIP1, Src homology 2 domain–containing inositol polyphosphate 5-phosphatase 1; TIRF-M, total internal reflection fluorescence microscopy.

Based on our HDX-MS results and SHIP1 structure predictions, we hypothesize that the SH2–C2 interaction would partially occlude membrane binding in the presence of PS and PI(3,4,5)P_3_ lipids (Fig. 5*A*). To test for this mechanism, we measured the membrane binding frequency of various mNG-tagged SHIP1 constructs on supported membranes using single-molecule TIRF-M (Fig. 5, *B* and *C*). Consistent with the SH2 blocking the C2 domain–mediated interaction with PS lipids, the cumulative membrane binding frequency of autoinhibited mNG-mSHIP1(ΔCTD) was 4.7-fold lower compared with mNG-SHIP1(PH-PP-C2) measured at the same protein concentration (Figs. 5*D* and S6). By contrast, mNG-mSHIP1(ΔCTD) containing either the 3D or 7A mutations in the CBL1 motif displayed an increased number of membrane binding events compared with the WT enzyme (Fig. 5*D*). By measuring the membrane binding frequency over a range of protein concentrations, we calculated the association rate constant (*k*_ON_) for each mNG-SHIP1 construct (Fig. S6). The mNG-mSHIP1(ΔCTD) construct has an 8.3-fold lower *k*_ON_ compared with mNG-SHIP1(PH-PP-C2) (Fig. 5*E*). When measuring the *k*_ON_ for mNG-mSHIP1(ΔCTD) 3D and 7A mutants, we observed an intermediate association rate constant that was 3.3- and 3.4-fold higher compared with WT mNG-mSHIP1(ΔCTD). This indicates that the CBL1 motif mutations reduce the ability of the SH2 domain to sterically hinder the C2 domain from membrane binding. Since mNG-SHIP1(PH-PP-C2) and mNG-mSHIP1(ΔCTD) differ in molecular weight and overall structure, they are predicted to have slightly different membrane binding frequency based on diffusion through solution. Importantly, the membrane binding frequency of the mNG-mSHIP1(ΔCTD) 3D and 7A mutants were nearly identical, indicating that the net charge of the CBL1 motif does not strongly influence the membrane binding frequency.

### Dimerization modulates SHIP1 membrane binding and apparent phosphatase activity

**Figure 6:**
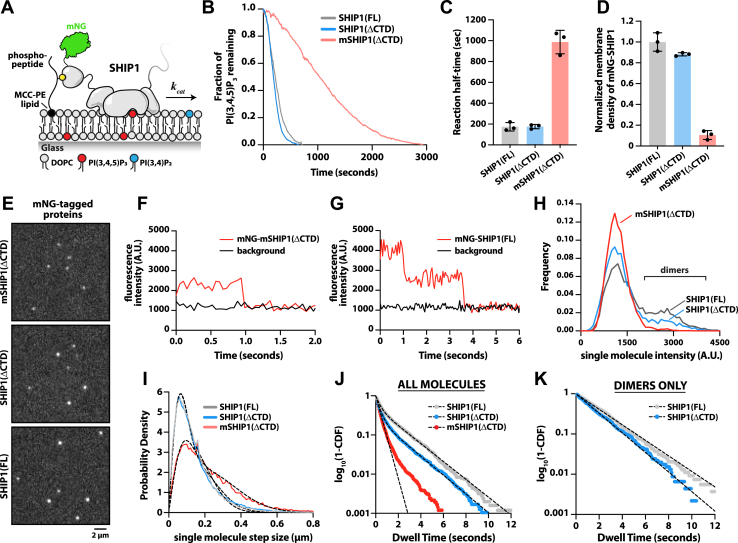
**Dimerization enhances SHIP1 membrane recruitment and apparent phosphatase activity.***A*, cartoon illustrating experimental design for visualizing membrane recruitment of mNG-SHIP1 to pY peptides. *B*, representative kinetic traces of SHIP1 phosphatase activity in the presence of 100 pM mNG-SHIP1(FL), mNG-SHIP1(ΔCTD), or mNG-mSHIP1(ΔCTD). All reactions contained 20 nM Btk-SNAP-AF546 to measure dephosphorylation of PI(3,4,5)P_3._*C*, quantification of reaction half-times for mNG-SHIP1(FL), mNG-SHIP1(ΔCTD), and mNG-mSHIP1(ΔCTD) in the presence of coupled pY-ITIM peptides (N = 3 technical replicates per construct). *D*, normalized mNG-SHIP1 membrane surface density from reactions in (*B*). Fluorescence intensity of different mNG-SHIP1 constructs was normalized relative to the average membrane intensity of mNG-SHIP1(FL). *E*, representative TIRF-M imaging showing the single-molecule localization of mNG-SHIP1 proteins (20–50 pM solution concentration). *F* and *G*, single-molecule photobleaching dynamics of (*F*) mNG-mSHIP1(ΔCTD) and (*G*) mNG-SHIP1(FL) compared with background fluorescence (*black line*). *H*, molecular brightness distributions measured in the presence of 50 pM mNG-SHIP1(FL), 50 pM mNG-SHIP1(ΔCTD), or 20 pM mNG-mSHIP1(ΔCTD). *I*, step size distribution plots generated from single-molecule tracking in the presence of either 50 pM mNG-SHIP1(FL), 50 pM mNG-SHIP1(ΔCTD), or 20 pM mNG-mSHIP1(ΔCTD). *J*, single-molecule dwell time distributions for all mNG-SHIP1 membrane binding events. Dwell times were calculated by fitting log_10_(1-cumulative distribution frequency [CDF]) to either a single or a double exponential decay curve (*black dashed lines*). *K*, single-molecule dwell time distributions for all mNG-SHIP1 membrane binding events for only the legitimate dimers observed in the presence of either mNG-SHIP1(FL) or mNG-SHIP1(ΔCTD). *B*–*K*, initial membrane composition: 2% PI(3,4,5)P_3_, 2% MCC-PE (+pY-ITIM peptide), 96% DOPC. Refer to Table 1 for statistics. Btk, Bruton’s tyrosine kinase; CTD, C-terminal domain; DOPC, 1,2-dioleoyl-*sn*-glycero-3-phosphocholine ; FL, full length; MCC-PE, 1,2-dioleoyl-*sn*-glycero-3-phosphoethanolamine-*N*-[4-(p-maleimidomethyl)cyclohexane-carboxamide; mNG, mNeonGreen; PI(3,4,5)P_3_, phosphatidylinositol-(3,4,5)-trisphosphate; pY, phosphotyrosine; SHIP1, Src homology 2 domain–containing inositol polyphosphate 5-phosphatase 1; TIRF-M, total internal reflection fluorescence microscopy.

Since mSHIP1(ΔCTD) lacks the dimerization domain, we hypothesized that differences in oligomerization could modulate SHIP1 membrane localization and phosphatase activity. To mimic the physiological context of SHIP1 membrane signaling, we measured SHIP1 activity and localization on SLBs containing membrane-conjugated pY-ITIM peptides (Fig. 6*A*). For these experiments, we simultaneously monitored the dephosphorylation of PI(3,4,5)P_3_ and the membrane density of various mNG-tagged SHIP1 proteins. The activity of mSHIP1(ΔCTD) was significantly reduced compared with FL SHIP1 and the SHIP1(ΔCTD) mutant (Fig. 6, *B* and *C*). Quantification of mNG fluorescence revealed that the difference in phosphatase activity could be explained by the enhanced membrane localization of mNG-SHIP1(FL) and mNG-SHIP1(ΔCTD), compared with mNG-mSHIP1(ΔCTD) (Fig. 6*D*). Considering the difference in membrane localization between the various SHIP1 constructs, the phosphatase activity per SHIP1 molecule is nearly identical, whereas the apparent activities are significantly different at the same protein solution concentration.

To quantify the abundance of SHIP1 dimers on membranes, we performed single-molecule TIRF-M experiments to visualize mNG-labeled SHIP1 (Fig. 6*E*, Movie S2). We previously used this approach to quantify differences in the oligomerization state of type I phosphatidylinositol-4-phosphate 5-kinases and a type II phosphatidylinositol-5-phosphate 4-kinase bound to phosphatidylinositol-(4,5)-bisphosphate lipids (33). Using SLBs with conjugated pY peptides (Fig. 6*A*), we performed single-molecule photobleaching experiments to determine the oligomerization state of membrane-bound mNG-tagged SHIP1 proteins. In the case of mNG-mSHIP1(ΔCTD), membrane-bound particles photobleached in a single step (Fig. 6*F*). By contrast, a subpopulation of mNG-SHIP1(FL) and mNG-SHIP1(ΔCTD) particles displayed two-step photobleaching events (Fig. 6*G*). We then compared the single-molecule brightness distributions for mNG-SHIP1 proteins bound to pY peptides. This analysis revealed the following fraction of legitimate dimers with two definitive mNG proteins visible: 1.7 ± 0.1% mNG-mSHIP1(ΔCTD), 13 ± 1.4% mNG-SHIP1(ΔCTD), and 24 ± 1.8% mNG-SHIP1(FL) (Fig. 6*H*). Considering that the degree of mNG chromophore maturation for recombinant proteins is approximately 75%, the maximum number of SHIP1 dimers we expect to observe is about 50% of the membrane-bound particles. Since the fraction of observed SHIP1 dimers was lower than predicted for a constitutively dimeric protein, mNG-SHIP1(FL) and mNG-SHIP1(ΔCTD) appear to exist in a monomer–dimer equilibrium under our experimental conditions. If we assume that the membrane binding frequency of monomeric and dimeric mNG-SHIP1 is equivalent, we estimate that 48% of FL mNG-SHIP1 and 26% of mNG-SHIP1(ΔCTD) exist as dimers under the experimental conditions used for single-molecule imaging (*i.e.*, pM concentrations). Note that the fraction could be greater in the presence of a higher solution concentration of mNG-SHIP1.

Oligomerization of peripheral membrane–binding proteins typically increases the frictional drag coefficient, which reduces membrane diffusivity (33, 34, 35). Comparing the single-molecule displacement across all trajectories revealed significantly slower membrane diffusivity for mNG-SHIP1(FL) and mNG-SHIP1(ΔCTD) compared with mNG-mSHIP1(ΔCTD) (Fig. 6*I* and Table 1). Consistent with the RBD-mediating dimerization, we also observed significantly longer membrane dwell times for mNG-SHIP1(FL) and mNG-SHIP1(ΔCTD) compared with mNG-mSHIP1(ΔCTD) (Fig. 6*J* and Table 1). The dwell time distribution for mNG-mSHIP1(ΔCTD) could be fit using a single exponential (τ_1_ = 0.41 s), whereas the SHIP1 constructs containing the RBD were best fit with a double exponential, yielding two characteristic dwell times (τ_1_ and τ_2_; Fig. 6*J* and Table 1). Interestingly, the fraction of long dwelling (τ_2_) mNG-SHIP1(FL) and mNG-SHIP1(ΔCTD) molecules was greater than the fraction of dimers calculated based on molecular brightness (Table 1; 1–α). As described previously, this is consistent with our predicted number of dimers after considering the incomplete maturation of the mNG. Quantification of the dwell time of only legitimate mNG-SHIP1(FL) and mNG-SHIP1(ΔCTD) dimers (*i.e.*, two mNG visible) yielded a single time constant (τ_1_) similar to the long dwelling species (*i.e.*, τ_2_) observed in the entire distribution of membrane binding events (Fig. 6*K* and Table 1). Together, this suggests that the fraction of long-dwelling mNG-SHIP1(FL and ΔCTD) molecules is a good proxy for the total number of dimer membrane binding events at equilibrium, which is 25% to 50%.

## Discussion

To decipher the mechanism of SHIP1 autoinhibition, we created a minimal SHIP1 construct that displays the primary characteristics of FL-autoinhibited SHIP1. This is akin to the approach used to determine the molecular basis of N-WASP autoinhibition, coincidence detection, and activation in the context of actin filament nucleation (36, 37). HDX-MS experiments performed with mSHIP1(ΔCTD) revealed that the SH2 domain shields the CBL1/3 motifs in the C2 domain, suggesting a mechanism for autoinhibition. Importantly, similar interdomain interactions were detected in the absence and presence of the dimerization motif (*i.e.*, RBD), which is positioned between the SH2 domain and central catalytic core (*i.e*., PH-PP-C2). Based on single-molecule TIRF-M measurements of the membrane binding frequency, autoinhibition reduces the ability of SHIP1 to dock on membranes containing anionic lipids. Mutations in the CBL1 motif, including charge swaps (K741D/K743D/K747D), were sufficient to relieve autoinhibition and increase the phosphatase activity of mSHIP1(ΔCTD). Complete charge neutralization of the CBL1 motif (*i.e*., 7A mutation) similarly increased the activity and membrane binding of mSHIP1(ΔCTD), indicating that the net charge of the CBL1 motif is not critical for membrane association. Our single-molecule TIRF-M measurements indicate that mutations in the CBL1 motif increase the membrane binding frequency of SHIP1 by reducing steric hindrance by the SH2 domain. Although the SHIP1 CBL1 motif is slightly divergent from SHIP2, there is a conservation of basic residues, which suggests that a similar autoinhibitory mechanism exists in SHIP2.

Mutations in the CBL1 motif had no effect on the PS lipid–enhanced activity measured in the context of the central catalytic core referred to as SHIP1(PH-PP-C2). In addition, CBL1 motif mutants did not perturb the membrane dwell time and diffusivity of mNG-SHIP1(PH-PP-C2) measured on SLBs and the plasma membrane of neutrophil-like cells. This suggests that the CBL1 motif modulates SHIP1’s activity through interactions with the SH2 domain and does not strongly influence PS binding. SHIP1/2 contains a P-variant (or type II) C2 domain, which is based on the orientation of β-sheets relative to the N- and C-terminal connections (38, 39). This naming convention, however, does not inform the function or specificity of the C2 domain. Unlike other lipid-binding domains (*e.g*., PH domains), C2 domains generally lack a conserved binding pocket or basic residues that confer lipid specificity. C2 domains also have low primary amino acid sequence conservation (40). Although many type-II C2 domains bind calcium, SHIP1 and SHIP2 lack conserved acidic residues in their CBL motifs to coordinate calcium, which mediates PS–lipid interactions. Consistent with these sequence characteristics, SHIP2 lipid binding and phosphatase activity are reportedly insensitive to calcium (23). Single-molecule membrane binding measurements similarly revealed no calcium dependence for SHIP1 (24). In general, the C2 domains of SHIP1/2 associate with anionic lipids in a calcium-independent manner. To date, no SHIP1 separation of function mutants (*e.g*., point mutations) have been rationally designed to eliminate the reported interactions between the C2 domain and anionic lipids. Without definitive structural biochemistry data, the molecular basis of SHIP1 specificity for PS lipid binding outside the catalytic phosphatase domain remains unclear.

Our HDX-MS experiments detected pY-mediated solvent exposure of residues in the CBL3 loop but only in the context of the dimeric SHIP1(ΔCTD) construct. Although the CBL3 motif might partially contribute to autoinhibition, charge reversal mutations in the CBL1 motif were sufficient to disrupt SHIP1 autoinhibition *in vitro*. Mutating the entire CBL1 motif (*i.e*., 7A mutant) also activates the minimal SHIP1 construct, mSHIP1(ΔCTD). By contrast, the charge neutralization mutations in the CBL1 motif (*i.e*., K741A/K743A/K747A) were unable to relieve autoinhibition. This could be due to the SH2 domain still having a sufficiently strong interaction with the C2 domain to mediate autoinhibition in the presence of the K741A/K743A/K747A mutations. There are several polar residues in the CBL1 motif that potentially hydrogen bond with the SH2 domain. Alternatively, there could be additional interactions between the SH2 domain and the CBL3 motif that facilitate autoinhibition in the context of the mSHIP1(ΔCTD) 3A mutant. Considering that the CBL1 and CBL3 motifs have opposite net charge, they could also interact with each other to form a surface that associates with the SH2 domain.

*In vivo*, alternative splicing of SHIP1/2 mRNA transcripts produces enzymes that lack the N-terminal SH2 domain (19, 41). Previous biochemical analysis indicates that these splice variants lack autoinhibition (24). The degree to which SHIP1 activity is modulated during cell signaling, however, will ultimately depend on cell type–specific expression of receptors and peripheral membrane–binding proteins that interact with either the N or C terminus of SHIP1. If cells contain an abundance of pY binding or SH3 domain–containing proteins that regulate SHIP1 localization *via* CTD interactions, it is expected that membrane-localized SHIP1(ΔSH2) will exhibit more phosphatase activity. By contrast, cells that primarily rely on receptor tyrosine kinases to regulate SHIP1 plasma membrane localization will likely exhibit sustained PI3K signaling because of the reduced ability of a SHIP1(ΔSH2) splice variant to localize to the plasma membrane.

**Figure 7:**
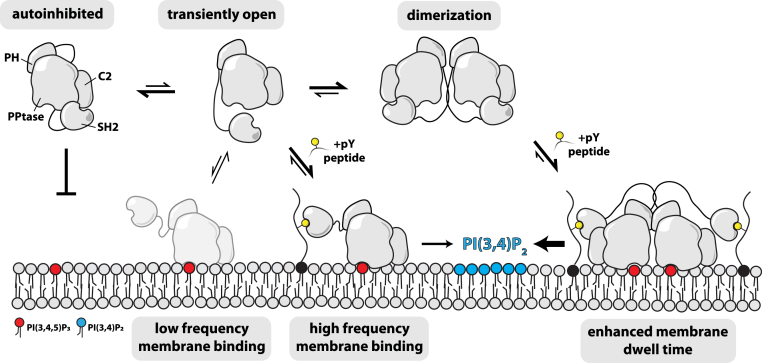
**Mechanisms controlling SHIP1 membrane localization and phosphatase activity.** In the autoinhibited state, the probability of SHIP1 membrane association is relatively low. Transient dissociation of the SH2–C2 domain interaction increases the probability of SHIP1 binding to anionic lipids. SHIP1 membrane interaction based solely on lipid interactions results in transient membrane dwell times. The presence of membrane tethered phosphotyrosine peptides enhances membrane localization and apparent phosphatase activity due to the increase in membrane resident time of SHIP1. Dimerization increases the membrane binding frequency and dwell time of SHIP1. This results in higher apparent phosphatase activity due to the increased membrane surface density observed in the presence of dimeric SHIP1. SH2, Src homology 2; SHIP1, Src homology 2 domain–containing inositol polyphosphate 5-phosphatase 1.

Overall, SHIP1 autoinhibition is regulated by weak intramolecular interactions between the N-terminal SH2 domain and the C2 domain. Although autoinhibitory interactions might restrict conformational changes in the phosphatase and C2 domains, our data suggest that SH2-mediated steric occlusion of the C2 domain functions to limit SHIP1 membrane association. As a result, SHIP1 activation is more dependent on tyrosine phosphorylated peptides, which are produced at the plasma membrane during cell signaling. Our HDX-MS data and structure predictions also suggest that the SH2–C2 domain interaction does not directly block the active site. Although the linker connecting the N-terminal SH2 domains and central catalytic core could limit substrate accessibility to the active site, we did not observe a statistically significant change in solvent exposure across the linker sequence in the presence of pY peptides. Our results are more consistent with the N-terminal SH2 domain forming weak intramolecular interactions with the C2 domain that reduce the membrane binding frequency. Interactions with tyrosine-phosphorylated peptides favor an open conformation of SHIP1. Dimerization of SHIP1 enhances membrane resident time by increasing the probability of the pY peptide engagement, resulting in high apparent phosphatase activity. Interaction with tyrosine phosphorylated peptides also increases the probability of collisions between anionic lipids and the PH-PP-C2 module, which promotes stable membrane docking and catalysis by SHIP1 (Fig. 7). This work provides a framework for future investigation of regulatory mechanisms, including small-molecule drug design, that modulate SHIP1 localization and activity by strengthening or weakening the SH2–C2 interface.

## Experimental procedures

### Molecular biology

The plasmid containing the open reading frame for human SHIP1 (or INPP5D) was purchased from Horizon Discovery (PerkinElmer), formerly known as Open Biosystems and Dharmacon. This SHIP1 clone represents isoform 2 (1–1189 amino acids) and is missing V117, located in the disordered linker that follows the N-terminal SH2 domain (UniProt: Q92835, isoform 2). Gene fragments containing SHIP1 were cloned into FAST Bac1 or pETM vectors using Gibson Assembly (42). Each protein expression construct was screened for optimal yield and solubility using either bacteria (BL21 DE3 Star, Rosetta, etc.) or insect cells. Lentiviral packing vectors were purchased from Addgene. The psPAX2 plasmid was a gift from Didier Trono (Addgene plasmid #12260; http://n2t.net/addgene:12260; Research Resource Identifier: Addgene_12260). The pVSV-G plasmid was a gift from Akitsu Hotta (Addgene plasmid #138479) (43). All plasmids were sequenced by Azenta/Genewiz and Plasmidsaurus to ensure open reading frames lacked deleterious mutations. Refer to the *Supporting Information* section for the complete list of plasmids used in this study.

### Protein purification

#### mNG-mSHIP1(ΔCTD) (WT, 3D, 3A, and 7A) and mNG-nSH2(PI3K)-SHIP1(PH-PP-C2)

The coding sequence of mSHIP1(ΔCTD) constructs was cloned into FASTBac1 vector with an N-terminal his6-tobacco etch virus (TEV)-mNG tag. Refer to the *Supporting Information* section for the exact amino acid sequences. BACMID DNA and baculovirus were generated as previously described (24, 33). High-five insect cells were infected with baculovirus at 2% v/v and grown for 48 h at 27 °C in ESF 921 Serum-Free insect cell culture medium (Expression Systems, catalog no.: 96-001-01). Cells were harvested by centrifugation at 3500 rpm for 10 min and transferred into 50 ml conicals using 1× PBS (pH 7.2) to resuspend and wash the cells. The cells were pelleted and resuspended in an equal volume of 1× PBS (pH 7.4) containing 10% glycerol and 2× protease inhibitors (one Sigma protease inhibitor tablet per 50 ml of buffer). The pellets were frozen down in a −80 °C until purification. To purify SHIP1 proteins, insect cell pellets were thawed in room temperature water and resuspended in buffer containing 30 mM Tris (pH 8.0), 10 mM imidazole, 5% glycerol, 400 mM NaCl, 1 mM PMSF, 2 mM beta-mercaptoethanol (BME), 1 Sigma protease inhibitor tablet per 100 ml of buffer, and 100 μg/ml DNase. Cells were lysed using the Dounce homogenizer. The lysate was centrifuged at 35,000 rpm at 4 °C in a Beckman Ti45 rotor. High-speed supernatant (HSS) was pooled and batch bound to 5 to 10 ml of nickel (Ni)–nitrilotriacetic acid (NTA) agarose resin (Qiagen, catalog no.: 30230) at 4 °C for 2 h, stirring in a beaker. The HSS and resin were poured into 50 ml conical tubes, centrifuged, and then washed with buffer containing 20 mM Tris (pH 8.0), 30 mM imidazole, 400 mM NaCl, 5% glycerol, and 2 mM BME. The resin bound to his6-mNG-mSHIP1(ΔCTD) constructs was transferred to a gravity flow column and washed with at least 100 ml of additional wash buffer. The protein was then eluted with buffer containing 20 mM Tris (pH 8.0), 300 mM imidazole, 400 mM NaCl, 5% glycerol, and 2 mM BME. Peak fractions were pooled, and his6-TEV protease (poly-E tail) was added to the elution to cleave off the polyhistidine tag. SHIP1 proteins were dialyzed overnight into a 4 l reservoir of buffer containing 20 mM Tris (pH 8.0), 150 mM NaCl, and 2 mM BME to reduce the salt concentration for ion exchange chromatography. The next day, the dialysate was transferred to a 50 ml tube and centrifuged at 3500 rpm at 4 °C to clarify. The supernatant was passed through a 0.22 μm syringe filter to remove any precipitation. Buffer containing 20 mM Tris (pH 8.0), 1 mM Tris(2-carboxyethyl)phosphine hydrochloride (TCEP) is added to the dialysate in a 1:2 ratio to lower the salt concentration to 100 mM NaCl. The protein was then bound to a 1 ml HiTrap CaptoQ anion exchange column (Cytiva, catalog no.: 11-0013-02) equilibrated in 20 mM Tris (pH 8.0), 100 mM NaCl, and 1 mM TCEP. Proteins were eluted with a linear salt gradient from 0.1 to 1 M NaCl over 22.5 min at a flow rate of 1.5 ml/min. The mNG-mSHIP1(ΔCTD) constructs elute at 300 to 450 mM NaCl. Peak fractions were pooled and concentrated in a 100 kDa molecular weight cutoff (MWCO) Amicon Ultra-4 spin concentrator (Sigma Millipore, catalog no.: UFC810024) to 0.5 to 1 ml. The protein was then loaded onto a 24 ml Superdex 200 10/300 GL (GE Healthcare, catalog no.: 17-5174-01) size-exclusion chromatography column equilibrated in buffer containing 20 mM Tris (pH 8.0), 150 to 200 mM NaCl, 10% glycerol, and 1 mM TCEP. The peak fractions were pooled and concentrated using a 100 kDa MWCO Amicon Ultra-4 spin concentrator. Proteins were aliquoted and flash frozen at a concentration of 1 to 30 μM using liquid nitrogen. The purification protocol for mSHIP1(ΔCTD), WT, and mutants was identical. Biophysical parameters of mNG-mSHIP1(ΔCTD): pI = 6.37, ε_280_ = 131,100 M^−1^ cm^−1^, 112.9 kDa. Biophysical parameters for mNG-PI3K(nSH2)-SHIP1(PH-PP-C2): pI = 6.17, ε_280_ = 138,090 M^−1^ cm^−1^, 115 kDa.

#### SHIP1 (FL and ΔCT)

The coding sequence of FL human SHIP1 (1–1189 amino acids) was cloned into a modified FastBac1 vector containing an N-terminal his6-TEV or his6-TEV-mNG fusion. High-five insect cells were infected with baculovirus using an optimized multiplicity of infection, typically 2% v/v, which we empirically determined based on small-scale test expressions (25–50 ml culture). Infected cells were typically grown for 48 h at 27 °C in ESF 921 Serum-Free insect cell culture medium (Expression Systems, catalog no.: 96-001-01). Cells were harvested by centrifugation, transferred to 50 ml tubes by washing with 1× PBS (pH 7.4), pelleted and resuspended in 10 ml of 1× PBS (pH 7.4), 10% glycerol, and 2× protease inhibitor cocktail (one Sigma protease inhibitor tablet per 50 ml of buffer) and then stored in the −80 °C freezer. For purification, frozen cell pellets were thawed in an ambient water bath and lysed into buffer containing 30 mM Tris (pH 8.0), 10 mM imidazole, 5% glycerol, 400 mM NaCl, 1 mM PMSF, and 2 mM BME, Sigma protease inhibitor cocktail EDTA-free per 100 ml lysis buffer, and 100 μg/ml DNase using the Dounce homogenizer. Lysate was then centrifuged at 35,000 rpm for 60 min in a Beckman Ti 45 (catalog no.: 339160) rotor at 4 °C. HSS was then batch bound to 5 ml of Ni–NTA agarose (catalog no.: 30230) resin at 4 °C for 2 h, stirring in a beaker. The resin and HSS were collected in 50 ml tubes, centrifuged, and washed with buffer containing 20 mM Tris (pH 8.0), 30 mM imidazole, 300 to 400 mM NaCl, 5% glycerol, and 2 mM BME before being transferred to a gravity flow column. Ni–NTA resin with bound his6-TEV-mNG-SHIP1 was then washed with an additional 100 ml of 20 mM Tris (pH 8.0), 30 mM imidazole, 300 to 400 mM NaCl, 5% glycerol, and 2 mM BME buffer and then eluted into buffer containing 300 mM imidazole. Peak fractions were pooled and desalted using a G25 Sephadex desalting column (GE Healthcare/Cytiva, catalog no.: 17-5087-01) equilibrated in 20 mM Tris (pH 8.0), 100 mM NaCl, and 1 mM TCEP buffer. Any precipitation was removed *via* 0.22 μm syringe filtration. Clarified SHIP1 fractions were then bound to a HiTrap 1 ml CaptoQ anion exchange column (Cytiva, catalog no.: 11-0013-02) equilibrated in 20 mM Tris (pH 8.0), 100 mM NaCl, and 1 mM TCEP buffer. Proteins were resolved over a 10% to 100% linear gradient (0.1–1 M NaCl, 30 min, 1.5 ml/min flow rate). His6-TEV-mNG-SHIP1 elutes in the presence of 200 to 400 mM NaCl. Peak fractions containing SHIP1 were pooled, incubated with 400 μl of 243 μM his6-TEV protease (S291V) with poly-R tail for ∼12 to 16 h at 4 °C, concentrated in a 100 kDa MWCO Amicon concentrator (Sigma Millipore), and then loaded onto a 24 ml Superdex 200 10/300 GL (GE Healthcare, catalog no.: 17-5174-01) size-exclusion column equilibrated in 20 mM Tris (pH 8.0) 200 mM NaCl, 10% glycerol, 1 mM TCEP, and 0.05% CHAPS buffer (SHIP1 [ΔCT] final buffer contained no CHAPS and 0.5 mM TCEP). Peak fractions were concentrated in a 100 kDa MWCO Amicon Ultra-4 spin concentrator and flash frozen at a final concentration of 1 to 30 μM using liquid nitrogen. Purification schemes for FL SHIP1, SHIP1(ΔSH2), and SHIP1(ΔCT) were identical. Biophysical parameters of FL SHIP1: pI = 7.37, ε_280_ = 106,690 M^−1^ cm^−1^, 133.3 kDa.

#### mNG-SHIP1(PH-PP-C2)

BL21 (DE3) Star bacteria were transformed with his10-TEV-mNG-GGGGG-SHIP1 (mNG-PH-PP-C2, 191–878 amino acids), WT, and mutant (K741D/K743D/K747D) and plated on LB agar containing 50 μg/ml kanamycin. The following day, 50 ml of TPM media (20 g tryptone, 15 g yeast extract, 8 g NaCl, 2 g Na_2_HPO_4_, and 1 g KH_2_PO_4_ per liter) containing 50 μg/ml kanamycin was inoculated with one bacterial colony from the agar plate. This culture was grown for 12 to 16 h at 27 °C to an absorbance of 2 to 3 at 600 nm, before being diluted to an absorbance of 0.05 at 600 nm in 2 l of TPM media. These cultures were grown at 37 °C to an absorbance of 0.8 at 600 nm, shifted to 30 °C for 1 h, and then bacteria were induced with 50 μM IPTG to express mNG-SHIP1(PH-PP-C2). After 6 to 8 h of expression at 30 °C, bacterial cultures were harvested by centrifugation, transferred to 50 ml tubes by washing with 1× PBS (pH 7.4), pelleted, and then stored in the −80 °C freezer. The PH-PP-C2 (K741D/K743D/K747D) protein was diluted into 2 l of TB media and expressed at 18 °C for 18 to 20 h before harvesting. For purification, frozen cell pellets were thawed in an ambient water bath and lysed into buffer containing 50 mM Na_2_HPO_4_ (pH 8.0), 400 mM NaCl, 1 mM PMSF, 0.4 mM BME, and 100 μg/ml DNase by microtip sonication (12–24 × 5 s pulses, 40% amplitude). Lysate was then centrifuged at 15,000 rpm (35,000*g*) for 60 min in a Beckman JA-20 rotor at 4 °C. During lysate centrifugation, a 5 ml HiTrap chelating column (Cytiva, catalog no.: 17040901) was charged with 50 ml of 100 mM CoCl_2_, generously washed with water (phosphate buffer will precipitate unchelated CoCl_2_ if not thoroughly removed), and equilibrated with lysis buffer lacking PMSF and DNase (>0.4 mM BME will destroy the charged HiTrap column and turn the column brown or black). HSS was then circulated over a charged 5 ml HiTrap column for 2 h. Captured protein was washed with 100 ml of 50 mM Na_2_HPO_4_ (pH 8.0), 400 mM NaCl, 10 mM imidazole, and 0.4 mM BME and then eluted into buffer containing 500 mM imidazole. Peak fractions containing SHIP1 were pooled with 400 μl of 243 μM his6-TEV protease (S291V) with poly-R tail and dialyzed in 4 l of 25 mM Na_2_HPO_4_ (pH 8.0), 400 mM NaCl, and 0.4 mM BME buffer at 4 °C for ∼12 to 16 h to remove imidazole. The next day, the charged HiTrap column was equilibrated with dialysis buffer, and dialyzed protein was recirculated for 2 h at 4 °C to remove his6-TEV protease and uncleaved his10-TEV-mNG-GGGGG-SHIP1(PH-PP-C2). Unbound protein was then concentrated in a 30 kDa MWCO Amicon concentrator (Sigma Millipore) and then loaded onto a 24 ml Superdex 200 10/300 GL (GE Healthcare, catalog no.: 17-5174-01) size-exclusion column equilibrated in 20 mM Tris (pH 8.0), 200 mM NaCl, 10% glycerol, and 1 mM TCEP buffer. Peak fractions were concentrated in a 30 kDa MWCO Vivaspin 6 centrifuge tube and flash frozen at a final concentration of 10 to 30 μM using liquid nitrogen. Biophysical parameters of mNG-SHIP1(PH-PP-C2): pI = 7.43, ε_280_ = 116,660 M^−1^ cm^−1^, 94.4 kDa.

#### mNG-SHIP1 (RBD)

BL21 (DE3) Star bacteria were transformed with his10-mNG-GGGGG-SHIP1 (RBD, 151–290 amino acids) and plated onto an LB agar plate containing 50 μg/ml kanamycin. To express the protein, 50 ml of TPM media with 50 μg/ml kanamycin was inoculated with one bacterial colony and grown at 27 °C for 12 to 16 h until an absorbance of 1.5 to 2 at 600 nm. The culture was then diluted into 2 l of TB media to an absorbance of 0.05 at 600 nm. The cultures were grown at 37 °C for 1.5 to 3 h until an absorbance reached 0.6 to 0.8 at 600 nm. The temperature was shifted to 18 °C for 1 h before inducing expression of mNG-SHIP1 (RBD) with 100 μM IPTG. The cultures were then allowed to grow for 16 to 20 h before harvesting. The bacterial cultures were harvested by centrifugation, and the pellets were resuspended in 1× PBS (pH 7.4) and transferred to 50 ml conicals for storage at −80 °C. Frozen cell pellets were thawed for purification in an ambient water bath and lysed into buffer containing 50 mM Na_2_HPO_4_ (pH 8.0), 400 mM NaCl, 1 mM PMSF, 0.4 mM BME, and 100 μg/ml DNase. The cells were then lysed by sonication (24 pulses each 5 s at 40% amplitude). The lysate was then centrifuged at 15,000 rpm at 4 °C for 1 h in JA-20 rotor (Beckman). The HSS was then circulated for 2 h over a HiTrap chelating column loaded with 50 ml 200 mM CoCl_2_ that was equilibrated in lysis buffer excluding PMSF and DNase. After circulation, the HiTrap column was washed with 100 ml of wash buffer containing 50 mM Na_2_HPO_4_ (pH 8.0), 400 mM NaCl, 10 mM imidazole, and 0.4 mM BME and then eluted into buffer with 500 mM imidazole. The peak fractions were pooled, and 400 μl of 250 mM his6-TEV protease was added to cleave the histidine tag and dialyzed into 4 l of 25 mM Na_2_HPO_4_ (pH 8.0), 400 mM NaCl, and 0.4 mM BME buffer overnight at 4 °C. The following morning, the dialysate was recirculated over the HiTrap column to trap the cleaved His tag and the TEV protease. The dialysate was then concentrated to ∼0.5 ml in 30 kDa MWCO Amicon concentrator (Sigma Millipore) to load on the Superdex200 10/300 GL column (GE Healthcare, catalog no.: 17-5174-01) equilibrated in buffer containing 20 mM Hepes (pH 7), 200 mM NaCl, 10% glycerol, and 1 mM TCEP. Peak fractions were determined by an absorbance at 280 nm and SDS-PAGE, and the fractions were pooled and concentrated using a 30 kDa MWCO Amicon concentrator (Sigma Millipore). The concentrated protein was aliquoted and flash frozen at a concentrated at 70 to 180 μM with liquid nitrogen and subsequently stored at −80 °C. Biophysical parameters of mNG-SHIP1(RBD): pI = 6.45, ε_280_ = 47,330 M^−1^ cm^−1^, and 42.7 kDa for the monomeric species.

#### Btk-SNAP

The PI(3,4,5)P_3_-binding domain derived from Btk was recombinantly expressed and purified as a his6-SUMO-Btk(PH-TH,R49S/K52S)-SNAP fusion protein using a protocol previously described (26). This construct contains mutations (*i.e*., R49S/K52S) in the peripheral lipid-binding domain, which eliminate dimerization and the valency of PI(3,4,5)P_3_–lipid interactions (25, 57). The his6-SUMO-Btk(PH-TH,R49S/K52S)-SNAP construct was recombinantly expressed in BL21 Star *Escherichia coli*. Bacteria were initially grown at 37 °C in terrific broth to an absorbance of 0.8 at 600 nm. Cultures were then shifted to 18 °C for 1 h, induced with 0.1 mM IPTG, and allowed to express protein for 20 h at 18 °C before being harvested. Cells were lysed into 50 mM NaPO_4_ (pH 8.0), 400 mM NaCl, 0.5 mM BME, 10 mM imidazole, and 5% glycerol. Lysate was then centrifuged at 16,000 rpm (35,172*g*) for 60 min in a Beckman JA-20 rotor chilled to 4 °C. Lysate was circulated over 5 ml HiTrap Chelating column (GE Healthcare, catalog no.: 17-0409-01), washed, and then eluted with linear gradient of lysis buffer containing 0 to 500 mM imidazole (8 CVs, 40 ml total, 2 ml/min flow rate). Peak fractions were pooled and combined with SUMO protease Ulp1 to cleave the his6-SUMO tag. The protein was dialyzed against 4 l of buffer containing 20 mM Tris (pH 8.0), 200 mM NaCl, and 0.5 mM BME for 18 h at 4 °C. The next day, cleaved Btk was recirculated for 1 h over a 5 ml HiTrap chelating column. Flow-through containing Btk-SNAP was then concentrated in a 5 kDa MWCO Vivaspin 20 before being loaded on a Superdex 75 size-exclusion column equilibrated in 20 mM Tris (pH 8.0), 200 mM NaCl, 10% glycerol, and 1 mM TCEP. Peak fractions containing Btk-SNAP were pooled and concentrated to a concentration of 30 μM before snap-freezing with liquid nitrogen and storage at −80 °C. For labeling, Btk-SNAP was combined with a 1.5× molar excess of SNAP-Surface Alexa546 dye (NEB, catalog no.: S9132S) and incubated overnight at 4 °C. The next day, Btk-SNAP-AF546 was desalted into buffer containing 20 mM Tris (pH 8.0), 200 mM NaCl, 10% glycerol, and 1 mM TCEP using a PD10 column. The protein was then concentrated using an Amicon filter and loaded onto a Superdex 75 column separate Btk-SNAP-AF546 from free AF546 dye. The peak elution was pooled, concentrated, aliquoted, and flash frozen with liquid nitrogen.

### Preparation of SLBs

SLBs were created to maximize the membrane fluidity at room temperature (*i.e.*, 23 °C). The membrane composition is primarily 1,2-dioleoyl-*sn*-glycero-3-phosphocholine (DOPC) (*i.e*., 94–98%), which has a melting temperature of −20 °C (44). Unless otherwise noted, SLBs also include PI(3,4,5)P_3_ as the substrate for SHIP1 and 20% PS to make the membrane more electronegative like the plasma membrane. In addition, some experiments include maleimide-functionalized lipids in order to conjugate pY peptides to the membrane. Although the SLBs lack complexity that exists on the plasma membrane *in vivo*, this experimental platform enables the SHIP1 phosphatase activity and membrane-binding interactions to analyze using a reductionist biochemistry approach.

The following lipids were used to generate small unilamellar vesicles (SUVs): 18:1 DOPC (Avanti, #850375C), 1,2-dioleoyl-*sn-*glycero-3-phospho-l-serine (18:1, Avanti, #840035C), 1,2-dioleoyl-*sn*-glycero-3-phosphoethanolamine-*N*-[4-(p-maleimidomethyl)cyclohexane-carboxamide (18:1 MCC-PE, Avanti, #780201C), and d-myo-PI(3,4,5)P_3_ (diC16, Echelon, P-3916-100 ug). To prepare SUVs, 2 μmol of total lipids were combined in a 35 ml glass round bottom flask containing 2 ml of chloroform. Lipids were dried to a thin film using rotary evaporation with the glass round-bottom flask submerged in a 42 °C water bath. After evaporating all the chloroform, the round bottom flask was place under a vacuum for 15 min. The lipid film was then resuspended in 2 ml of PBS (pH 7.4), making a final concentration of 1 mM total lipids. All lipid mixtures expressed as percentages (*e.g*., 98% DOPC, 2% PI(3,4,5)P_3_) are equivalent to molar fractions. For example, a 1 mM lipid mixture containing 98% DOPC and 2% PI(3,4,5)P_3_ is equivalent to 0.98 mM DOPC and 0.02 mM PI(3,4,5)P_3_. To generate 50 nm SUVs, 1 mM total lipid mixtures were extruded through a 0.05 μm pore size 19 mm polycarbonate membrane (Cytiva Whatman, catalog no.: 800308) with filter supports (Cytiva Whatman, catalog no.: 230300) on both sides of the polycarbonate membrane.

SLBs were formed on 25 × 75 mm coverglass (IBIDI, #10812) as previously described (33). In brief, coverglass was first cleaned with 2% Hellmanex III (ThermoFisher, catalog no.:14-385-864) and then etched with Pirahna solution (1:3, hydrogen peroxide:sulfuric acid) for 15 min. Etched coverglass, washed extensively with MilliQ water again, is rapidly dried with nitrogen gas before adhering to a 6-well sticky-side chamber (IBIDI, catalog no.: 80608). SLBs were formed by flowing 0.25 mM total lipid concentration of 50 nm SUVs diluted in 1× PBS pH (7.4) into an assembled IBIDI chamber. SUVs were incubated in the IBIDI chamber for 30 min and then washed with 4 ml of PBS (pH 7.4) to remove nonabsorbed SUVs. Membrane defects are blocked for 5 min with a solution of clarified 1 mg/ml beta casein (ThermoFisher, catalog no.: 37528) in 1× PBS (pH 7.4). After blocking membranes with 1 mg/ml beta casein for 5 min, bilayers were washed with 4 ml of 1× PBS. Reaction/imaging buffer was exchanged into the chamber prior to each experiment.

Conjugation of pY peptides to supported membranes was achieved as previously described (24). Supported membrane containing 2% MCC-PE lipids were used to covalently couple phosphorylated peptides (**C**KTEAENTIT(pY)SLIK, Elim Biopharmaceuticals) derived from the ITIM sequence FcγRIIB (45). A cysteine (bold) was added to the N terminus of the phosphorylated peptide to form a covalent bond with the maleimide lipid headgroup of the MCC-PE lipids. For membrane coupling, 100 μl of 10 μM pY-ITIM peptides diluted in a 1× PBS (pH 7.4) and 0.1 mM TCEP buffer was added to the IBIDI chamber and incubated for 2 h at 23 °C. Unreacted MCC-PE lipids were then quenched 15 min using 1× PBS (pH 7.4) with 5 mM BME. Quenched membranes were then washed and stored in 1× PBS (pH 7.4).

### Lipid phosphatase activity measurements

All 5-phosphatase activity measurements were performed using SHIP1 proteins with N-terminal mNG fusions. For simplicity, the mNG notation was omitted from all the graphs shown in Figures 1, 4, 5, S5. The kinetics of SHIP1-mediated dephosphorylation PI(3,4,5)P_3_ to generate PI(3,4)P_2_ was monitored on SLBs formed on piranha etched glass in an IBIDI chamber using TIRF-M. Phosphatase reactions contained 20 mM Hepes (pH 7.0), 100 mM NaCl, 1 mM ATP, 5 mM MgCl_2_, 0.5 mM EGTA, 200 μg/ml beta casein, 20 mM BME, 20 mM glucose, and 0.32 mg/ml glucose oxidase, 50 μg/ml catalase, and 1 mM Trolox. Membranes contained the following initial composition: 98% DOPC, 2% PI(3,4,5)P_3_, or 96% DOPC, 2% PI(3,4,5)P_3_, 2% MCC-PE or 78% DOPC, 20% 1,2-dioleoyl-*sn-*glycero-3-phospho-l-serine, and 2% PI(3,4,5)P_3_. Membrane compositions are indicated in the figure legends. All kinetic measurements utilized 20 nM Btk-SNAP-AF546 (PH domain; PI(3,4,5)P_3_ sensor) to visualize the dephosphorylation of PI(3,4,5)P_3_ by TIRF-M. Assuming a footprint of 0.72 nm^2^ for DOPC lipids, we calculated a density of 27,778 lipids/μm^2^ for 2% PI(3,4,5)P_3_ (46, 47).

### Oxygen scavenging system

Glucose oxidase (32 mg/ml, 100× stock) and catalase (5 mg/ml, 100× stock) were solubilized in 20 mM Hepes (pH 7.0), 150 mM NaCl, 10% glycerol, and 1 mM TCEP buffer, and then flash frozen in liquid nitrogen and stored at −80 °C. Trolox (200 mM, 100× stock) was UV treated and stored at −20 °C. Approximately 10 min before imaging, 100× oxygen scavenger stocks were diluted in kinase buffer containing enzymes/biosensors to achieve a final concentration of 320 μg/ml glucose oxidase (Serva, catalog no.: 22780.01, *Aspergillus niger*), 50 μg/ml catalase (Sigma, catalog no.: C40-100MG, bovine liver), and 2 mM Trolox (Cayman Chemicals, catalog no.: 10011659). Trolox was prepared using a previously described protocol that utilized UV irradiation to drive the formation of a quinone species (46, 48).

### HDX-MS sample preparation

HDX reactions comparing mNG-SHIP1(ΔCTD) ± 40 μM pY peptide were carried out in a 10 μl reaction volume containing 15 pmol (1.5 μM) of mNG-SHIP1(ΔCTD). The exchange reactions were initiated by the addition of 7.5 μl of D_2_O buffer (20 mM Hepes [pH 7.5], 200 mM NaCl, 0.5 mM TCEP, and 94.25% D_2_O [v/v]) to 2.5 μl of protein (final D_2_O concentration of 70.7%). Reactions proceeded for 3 s, 30 s, 300 s and 3000 s at 20 °C before being quenched with ice-cold acidic quench buffer, resulting in a final concentration of 0.6 M guanidine HCl and 0.9% formic acid post quench. HDX reactions comparing apo mNG-mSHIP1(ΔCTD) ± 40 μM pY peptide were carried out in 10 μl reaction volumes containing 15 pmol of protein. The exchange reactions were initiated by the addition of 7 μl of D_2_O buffer (20 mM Hepes [pH 7.5], 50 mM NaCl, and 95.24% D_2_O [v/v]) to 3 μl of protein (final D_2_O concentration of 66.67% [v/v], 1.5 μM mNG-mSHIP1(ΔCTD), and 40 μM pY peptide). Reactions proceeded for 3 s or 300 s before being quenched with ice-cold acidic quench buffer, resulting in a final concentration of 0.6 M guanidine HCl and 0.9% formic acid post quench. All conditions and timepoints were created and run in independent triplicate. Samples were flash frozen immediately after quenching and stored at −80 °C until injected onto the ultraperformance liquid chromatography (UPLC) system for proteolytic cleavage, peptide separation, and injection onto a QTOF for mass analysis, described later.

### Protein digestion and MS/MS data collection

Protein samples involving mNG-SHIP1(ΔCTD) were rapidly thawed and injected onto an integrated fluidics system containing a HDx-3 PAL liquid handling robot and climate-controlled (2 °C) chromatography system (LEAP Technologies), a Waters Acquity UPLC I-Class Series System, as well as an Impact HD QTOF Mass spectrometer (Bruker). mNG-mSHIP1(ΔCTD) protein samples were run using a Dionex Ultimate 3000 UHPLC system. The full details of the automated LC systems are previously described in Ref. (49). mNG-SHIP1(ΔCTD) samples were run over an immobilized pepsin column (Affipro; AP-PC-001) at 200 μl/min for 4 min at 2 °C. The resulting peptides were collected and desalted on a C18 trap column (Acquity UPLC BEH C18 1.7 μm column [2.1 × 5 mm]; Waters 186004629). The trap was subsequently eluted in line with an ACQUITY 300 Å, 1.7 μm particle, 100 × 2.1 mm BEH C18 UPLC column (Waters), using a gradient of 3% to 10% B (buffer A 0.1% formic acid; buffer B 100% acetonitrile) over 1.5 min, followed by a gradient of 10% to 25% B over 4.5 min, followed by a gradient of 25% to 35% B over 5 min, finally after 1 min at 35% B a gradient of 35% to 80% B over 1 min was used.

mNG-mSHIP1(ΔCTD) samples were run over one immobilized pepsin column (Trajan; ProDx protease column, 2.1 mm × 30 mm PDX.PP01-F32) at 200 μl/min for 3 min at 8 °C. The resulting peptides were collected and desalted on a C18 trap column (Acquity UPLC BEH C18 1.7 μm column [2.1 × 5 mm]; Waters 186003975). The trap was subsequently eluted in line with an ACQUITY 1.7 μm particle, 100 × 1 mm^2^ C18 UPLC column [Waters], using a gradient of 3% to 35% B (buffer A 0.1% formic acid; buffer B 100% acetonitrile) over 11 min immediately followed by a gradient of 35% to 80% over 5 min. MS experiments acquired over a mass range from 150 to 2200 *m/z* using an electrospray ionization source operated at a temperature of 200 °C and a spray voltage of 4.5 kV.

### Peptide identification

Peptides were identified from the nondeuterated samples of mNG-SHIP1(ΔCTD) using data-dependent acquisition following tandem MS/MS experiments (0.5 s precursor scan from 150 to 2000 *m/z*; 12 0.25 s fragment scans from 150 to 2000 *m/z*). MS/MS datasets were analyzed using FragPipe (Nesvizhskii lab, University of Michigan), v18.0, and peptide identification was carried out by using a false discovery–based approach using a database of purified proteins and known contaminants (50, 51, 52). MSFragger was utilized, and the precursor mass tolerance error was set to −20 to 20 ppm. The fragment mass tolerance was set at 20 ppm. Protein digestion was set as nonspecific, searching between lengths of 4 and 50 amino acids, with a mass range of 400 to 5000 Da. mNG-mSHIP1(ΔCTD) MS/MS datasets were analyzed using PEAKS7 (PEAKS), and peptide identification was carried out by using a false discovery-based approach, with a threshold set to 0.1% using a database of purified proteins and known contaminants. The search parameters were set with a precursor tolerance of 20 ppm, fragment mass error 0.02 Da, charge states from 1 to 8, leading to a selection criterion of peptides that had a −10logP score of 32.6.

### Mass analysis of peptide centroids and measurement of deuterium incorporation

HD-Examiner Software (Sierra Analytics) was used to automatically calculate the level of deuterium incorporation into each peptide. All peptides were manually inspected for correct charge state, correct retention time, appropriate selection of isotopic distribution, and so on. Deuteration levels were calculated using the centroid of the experimental isotope clusters. Results are presented as relative levels of deuterium incorporation, and the only control for back exchange was the level of deuterium present in the buffer (70.7% for mNG-SHIP1(ΔCTD) and 66.67% for mNG-mSHIP1(ΔCTD)). Differences in exchange in a peptide were considered significant if they met all three of the following criteria: ≥5% change in exchange, ≥0.4 Da difference in exchange, and a *p* value <0.01 using a two-tailed Student's *t* test for mNG-SHIP1(ΔCTD) experiment or ≥5% change in exchange, ≥0.4 Da difference in exchange, and a *p* value < 0.01 using a two-tailed Student's *t* test for mNG-mSHIP1(ΔCTD) experiment. Slight differences in significance thresholds between experiments is due to small differences in the system used for analysis. Samples were only compared within a single experiment and were never compared with experiments completed at a different time with a different final D_2_O level. The data analysis statistics for all HDX-MS experiments are provided in Tables S1 and S2 according to the published guidelines (53). The MS proteomics data have been deposited to the ProteomeXchange Consortium *via* the PRIDE partner repository (54) with the dataset identifier PXD058704 for mNG-SHIP1(ΔCTD) data and PXD061719 for mNG-mSHIP1(ΔCTD) data.

### Cell culture

PLB-985 neutrophil–like cells were grown in suspension in RPMI1640 + GlutaMAX media containing 25 mM Hepes (Life Technologies, catalog no.: 72400047), 9% fetal bovine serum (FBS), penicillin (100 units/ml) (Life Technologies, catalog no.: 15140122), and streptomycin (100 μg/ml) (Life Technologies, catalog no.: 15140122). These cells were provided by Professor Sean Collins (University of California at Davis) and were validated by RNA-Seq compared with primary human neutrophils (55). Cell lines were stored in a humidified incubator at 37 °C in the presence of 5% CO_2_ and split three times per week to maintain densities between 0.1 and 2 × 10^6^ cells/ml. PLB-985 cells were differentiated into a neutrophil-like state by culturing 0.2 × 10^6^ cells/ml for 6 to 7 days in RPMI media supplemented with 2% FBS, penicillin (100 units/ml), streptomycin (100 μg/ml), 1.3% dimethyl sulfoxide, and 2% Nutridoma-CS (Sigma, catalog no.: 11363743001). Nutridoma-CS was added to increase the chemotactic response of the cells.

Human embryonic kidney (HEK) 293T Lenti-X were obtained from Takara (catalog no.: 632180). These cells transformed with adenovirus type 5 DNA and expresses the SV40 large T antigen. HEK293T Lenti-X cells were cultured in Dulbecco's modified Eagle's medium (DMEM) + GlutMAX + high glucose (4.5 g/l) + sodium pyruvate (110 mg/l) (Life Technologies, catalog no.: 10569010) supplemented with 10% FBS (Sigma, catalog no.: F4135-500ML), penicillin (100 units/ml), and streptomycin (100 μg/ml). Cells were grown in 10 cm dishes in humidified incubators at 37 °C in the presence of 5% CO_2_ and split at a confluency of 80% to 90% every 2 to 3 days. HEK293T Lenti-X cells were split using 1.5 ml of Cellstripper (Corning, catalog no.: 25-056-Cl). Cellstripper was then quenched with 8.5 ml complete DMEM containing 10% FBS. Cells were diluted 1:10 and seeded on a new 10 cm dish containing a total volume of 10 ml complete DMEM warmed to 37 °C.

### Lentivirus production

Lentivirus was generated by transfecting 60% to 70% confluent HEK293 Lenti-X cells in a 10-cm plate with 6.7 μg psPAX2 (second-generation lentiviral packaging plasmid, Addgene #12260), 0.85 μg pVSV-G (expresses VSV-G envelope protein for pseudotyping NanoMEDIC particle, Addgene #138479) (43), 7.5 μg of transfer lentiviral vector containing gene of interest, and 30 μl of 1 mg/ml polyethyleneimine in 0.5 ml Opti-Mem (ThermoFisher, catalog no.: 31985070). This mixture was incubated for 15 min at room temperature before adding dropwise to plated HEK293 Lenti-X cells. Transfection was performed in complete media, and media were not changed at any point during the transection or lentiviral production process. Media containing lentivirus were harvested 48 h after beginning the transfection. Media containing lentivirus were clarified by centrifugation and then concentrated using Lenti-X concentrator (Takara, catalog no.: 631231). Precipitated virus was resuspended in 0.4 ml of complete RPMI media (9% FBS) and then stored at −80 °C. To transduce PLB-985 cells, 0.4 ml of concentrated lentivirus was added with 5 ml of undifferentiated cells at a density of 0.2 × 10^6^ cells/ml containing a final concentration of 8 μg/ml polybrene (Millipore, catalog no.: TR-1003-G, 10 mg/ml stock, 1250×) in the cell culture media. To increase transduction efficiency, spinoculation was performed in a 6-well dish spun at 1000*g* for 1 h at 33 °C. Infected PLB-985 cells were passaged at least one time before differentiating into the neutrophil-like state (see *Cell culture* for protocol).

### Live cell imaging

To image PLB-985 cells, 25 × 75 mm coverslips were cleaned overnight with 2% Hellmanex III (ThermoFisher, catalog no.: 14-385-864). The coverslips were rinsed well with MilliQ water and dried with nitrogen gas and attached to a 6-well IBIDI flow cell chamber (IBIDI sticky-Slide VI 0.4, catalog no.: 80608). Cells were imaged in Hank’s balanced salt solution (HBSS) containing 20 mM Hepes (pH 7.2), 150 mM NaCl, 4 mM KCl, 1 mM MgCl_2_, and 10 mM glucose that was warmed to 37 °C. A 10 μg/ml fibronectin (Sigma, catalog no.: F1141, 1 mg/ml stock concentration) solution diluted in 1× PBS was added to each well of the IBIDI chamber and incubated for 60 min at room temperature. Unbound fibronectin was washed out with 1× PBS and then blocked with 0.2% bovine serum albumin (fatty/endotoxin free, Sigma, catalog no.: B4501) dissolved in HBSS for 5 min. Differentiated PLB-985 (6 days postdifferentiation) were prepared for imaging by centrifuging 500 μl of cells at 100*g* for 10 min. The RPMI media were aspirated off, and the cells were resuspended in warm HBSS. The cells were flowed into the IBIDI chamber and allowed to adhere for 15 to 20 min. The cells were imaged under a Okolab-heated stage (catalog no.: H301-K-FRAME) warmed to 37 °C.

### Microscope hardware and imaging acquisition

Membrane binding and lipid dephosphorylation reactions reconstituted on SLBs were visualized using an inverted Nikon Eclipse Ti2 microscope using a 100× Nikon (1.49 numerical aperture) oil immersion TIRF objective. TIRF-M images of SLBs were acquired using an iXion Life 897 EMCCD camera (Andor Technology Ltd). Fluorescently labeled proteins were excited with either a 488 nm, 561 nm, or 637 nm diode laser (OBIS laser diode; Coherent, Inc) controlled with a Vortran laser drive with acousto-optic tunable filters control. The power output measured through the objective for single particle imaging was 1 to 2 mW. Excitation light was passed through the following dichroic filter cubes before illuminating the sample: (1) ZT488/647rpc and (2) ZT561rdc (ET575LP) (Semrock). Fluorescence emission was detected on the iXion Life 897 EMCCD camera position after a Nikon emission filter wheel housing the following emission filters: ET525/50M, ET600/50M, and ET700/75M (Semrock). All *in vitro* biochemistry experiments were reconstituted at room temperature (23 °C), whereas live cell imaging was performed at 37 °C using an Okolab-heated stage. Microscope hardware was controlled by Nikon NIS elements.

For single-molecule TIRF-M experiments involving mEos-SHIP1(PH-PP-C2), PLB-985 cells expressing low concentrations were identified based on 500 to 550 nm fluorescence emission following excitation with 488 nm light. Next, the red channel was imaged to confirm the lack of 575 to 625 nm emission before photoconversion. To photoconvert mEos from the green to red state, cells were globally excited with 1 to 3 mW of 405 light for 50 to 100 ms. To track association and dissociation of mEos-SHIP1(PH-PP-C2) at the plasma membrane, a 256 × 256-pixel region of interest was recorded on an iXion Life 897 EMCCD camera at 45 frames per second (*i.e*., 22 ms interval).

### Analysis of single-molecule TIRF-M data

Single particle detection and tracking was performed using the TrackMate plugin (56) on ImageJ/Fiji as previously described (33, 46). Output files are analyzed using Prism 9 graphing software. To calculate the single-molecule dwell times for fluorescently labeled SHIP1, we generated a cumulative distribution frequency plot using the frame interval as the bin size (*e.g*., 22 ms). The log_10_(1-cumulative distribution frequency) was plotted against the dwell time and fit with either a single or a double exponential decay curve.

Single-exponential model:

$$f\left(t\right)=e^{\left(-x/\tau \right)}$$

Two-exponential model:

$$f\left(t\right)={\alpha *e}^{\left(-x/\tau 1\right)}{+\left(1-\alpha \right)*e}^{\left(-x/\tau 2\right)}$$

Fitting procedure was initiated with a single exponential. In cases of a low-quality single exponential fit, a maximum of two populations were used. For double exponential fit, alpha (α) represents the percentage of short dwelling molecules characterized by the time constant, *τ*_1_. Errors reported for the single-molecule dwell times represent the standard deviation from multiple technical replicates as indicated in Table 1 and the figure legends.

To calculate the diffusion coefficients (μm^2^/s) for membrane-bound mNG-SHIP1(PH-PP-C2) in Fig. S7 and Table 1, we plotted probability density (*i.e*., frequency divided by bin size of 0.01 μm) *versus* step size (μm). The single-molecule displacement was measured over a 12 ms time interval with ∼20,000 displacements detected per technical replicate. Step size distributions were fit to the following two-species model:

$$f\left(r\right)=\alpha \frac{r}{2D_{1}\tau }e^{-\left(\frac{r^{2}}{4D_{1}\tau }\right)}+\left(1-\alpha \right)\frac{r}{2D_{2}\tau }e^{-\left(\frac{r^{2}}{4D_{2}\tau }\right)}$$

To calculate the cumulative membrane binding frequency in Figures 5 and S6, molecules were analyzed using the ImageJ/Fiji plugin, TrackMate. This allowed us to determine the exact frame when an mNG-SHIP1 molecule was first detected, which was then scored as a single membrane binding event. Although the program continues to track every molecule until membrane dissociation or photobleaching occurs, only new mNG-SHIP1 membrane docking events are scored and used to calculate the cumulative membrane binding frequency. All binding events for a single video were summed over a 30 s period, which represents cumulative membrane binding for a single mNG-SHIP1 concentration between 200 and 800 pM (Figs. 5*D* and S6).

To quantify the fraction of membrane-localized mNG-tagged SHIP1 monomers and dimers (Fig. 6), we wrote custom Python code extract the particle brightness in the second frame of each single-molecule trajectory. To ensure emitted photons were collected for the full duration of a 52 ms exposure, we measured the particle brightness in the second frame of tracks that were ≥156 ms (*i.e*., ≥3 frames). This approach is necessary because a molecule can associate or dissociate in the middle of the first or last frame of a trajectory. This results in fewer emitted photons and a lower observed molecular brightness. Particles were identified using TrackMate (ImageJ) using an object diameter of 7 pixels. The particle brightnesses reported in Figure 6*H* represent the maximum pixel intensity for each mNG-SHIP1 molecule detected.

## Data availability

All the information needed for interpretation of the data is presented in the article or the supporting information. Plasmids related to this work are available upon request.

## Supporting information

This article contains supporting information (figures [Figs. S1–S6], Movies S1-S2, tables, plasmid inventory, and peptide sequences (51, 52)).

## Conflict of interest

J. E. B. reports personal fees from Scorpion Therapeutics and Reactive Therapeutics and research contracts from OmniAb and Calico Life Sciences. All other authors declare that they have no conflicts of interest with the contents of this article.
