# Supplementary material for: SH2-mediated steric occlusion of the C2 domain regulates autoinhibition of SHIP1 inositol 5-phosphatase
Source: J Biol Chem. 2025 Oct 7;301(12):110788. doi: 10.1016/j.jbc.2025.110788 (PMC12657763; doi:10.1016/j.jbc.2025.110788)
Supplement: Supporting Information [file mmc1.pdf]

SUPPORTING INFORMATION

SH2-mediated steric occlusion of the C2 domain regulates autoinhibition of SHIP1 inositol 5-phosphatase

Emma E. Drew[1,2], Hunter G. Nyvall[3], Matthew A.H. Parson[3], Reed K. Talus[1,2], John E. Burke[3,4,5], and Scott D. Hansen[1,2*]

[1] Department of Chemistry and Biochemistry, University of Oregon, Eugene, OR 97403
[2] Institute of Molecular Biology, University of Oregon, Eugene, OR 97403
[3]Department of Biochemistry and Microbiology, University of Victoria, Victoria, British Columbia, V8W 2Y2, Canada
[4]Department of Biochemistry and Molecular Biology, The University of British Columbia, Vancouver, British Columbia V6T 1Z3, Canada
[5]University of Victoria Genome BC Proteomic Centre, Victoria, BC, Canada

*Corresponding author:
shansen5@uoregon.edu

Supplemental Figure 1

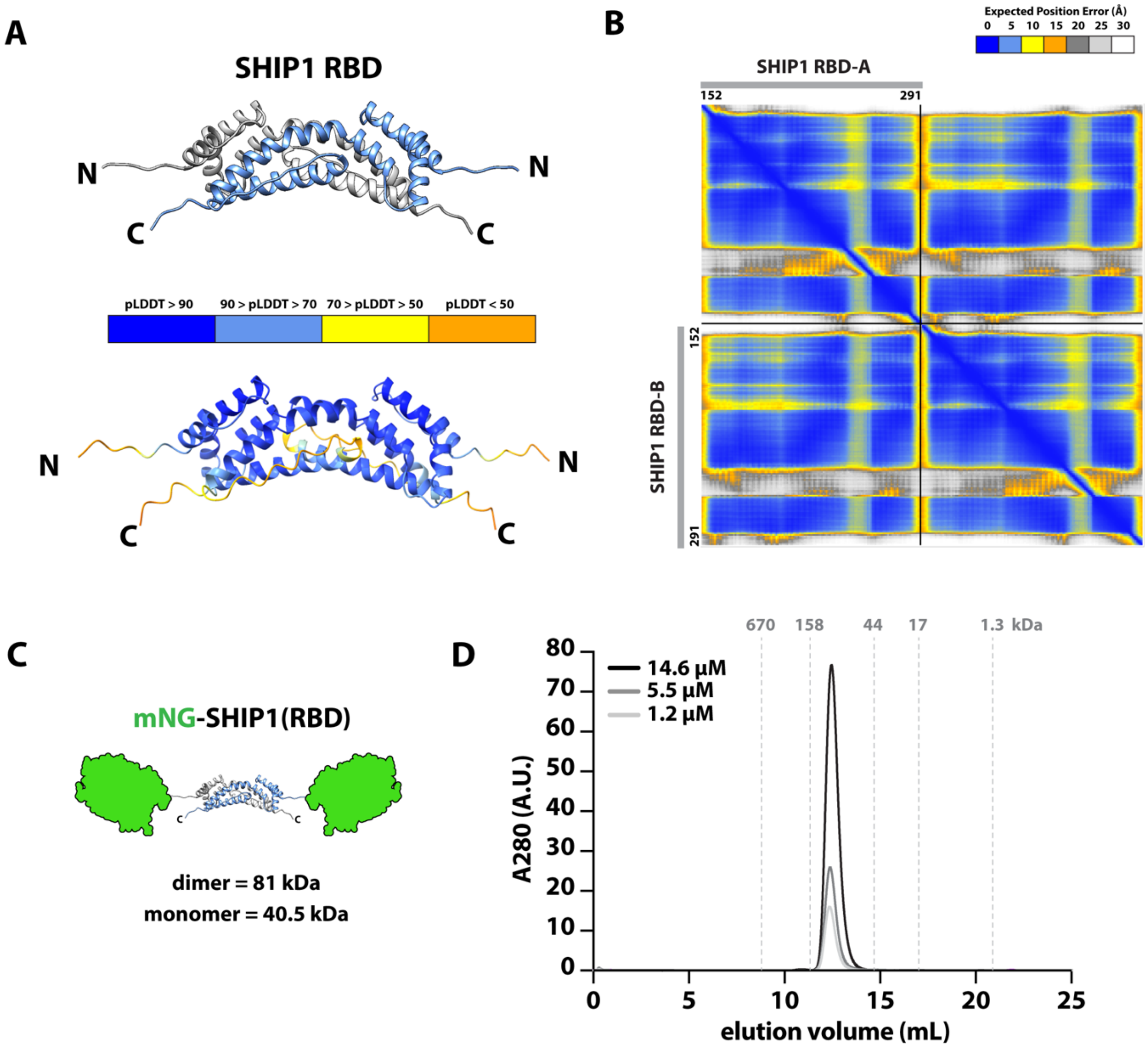

Figure Supplement 1

Characterization of SHIP1 Rho binding domain (RBD)

(A) AlphaFold2 Multimer prediction of the SHIP1 Rho binding domain (RBD) homodimerizing. Shown below is the model colored by predicted local distance difference (pLDDT) to show regions with high confidence (pLDDT > 90) to low confidence (pLDDT <50). **(B)** Predicted alignment error (PAE) for Alphafold2 multimer model of SHIP1 RBD dimer. Note that the PAE plot is not an inter-residue distance map or a contact map. Instead, the coloring indicates expected distance error. The color at (x, y) corresponds to the expected distance error in residue x's position (Angstroms), when the prediction are aligned on residue y (more information can be found at https://alphafold.ebi.ac.uk/). **(C)** Cartoon depiction of mNG-SHIP1(RBD) with the expected molecular weight of a dimer (81 kDa) or monomer (40.5 kDa). **(D)** Size exclusion chromatography elution profiles for varying concentration of mNG-SHIP1(RBD) injected on a Superdex200 Increase 10/300GL column (Cytiva, Cat# 28990944). Load concentration indicated in the legend. Elution profile of molecular weight standards (Bio-Rad, Cat#151-1901) indicated by grey dashed lines and kDa MW's above.

Supplemental Figure 2

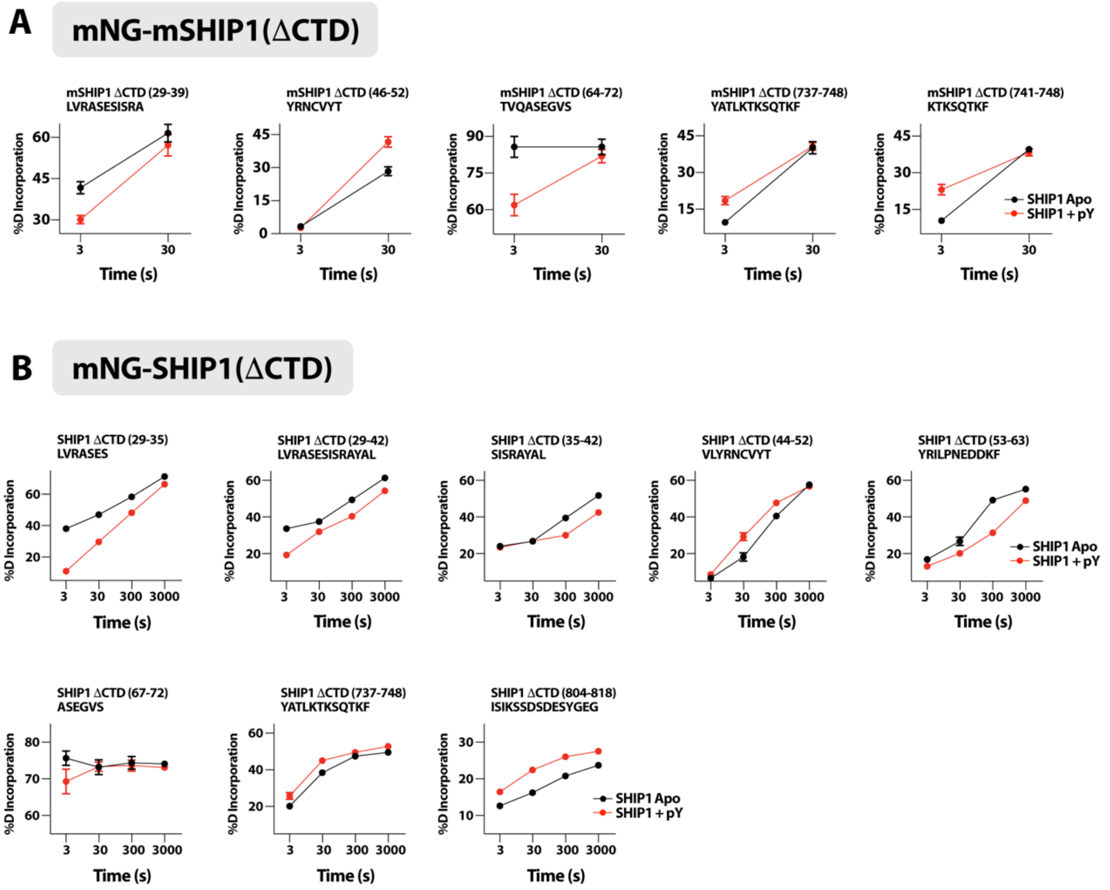

Figure Supplement 2
Percent deuterium incorporation graphs for SHIP1
Mean of the % deuterium uptake for **(A)** mNG-mSHIP1(ΔCTD) and **(B)** mNG-SHIP1(ΔCTD) peptides that showed a significant change in HDX (>0.4 Da and 5% difference, with a two-tailed t-test $p < 0.01$) across the entire deuterium exchange time course (error bars represent standard deviation, $n = 3$ for all timepoints).

Supplemental Figure 3

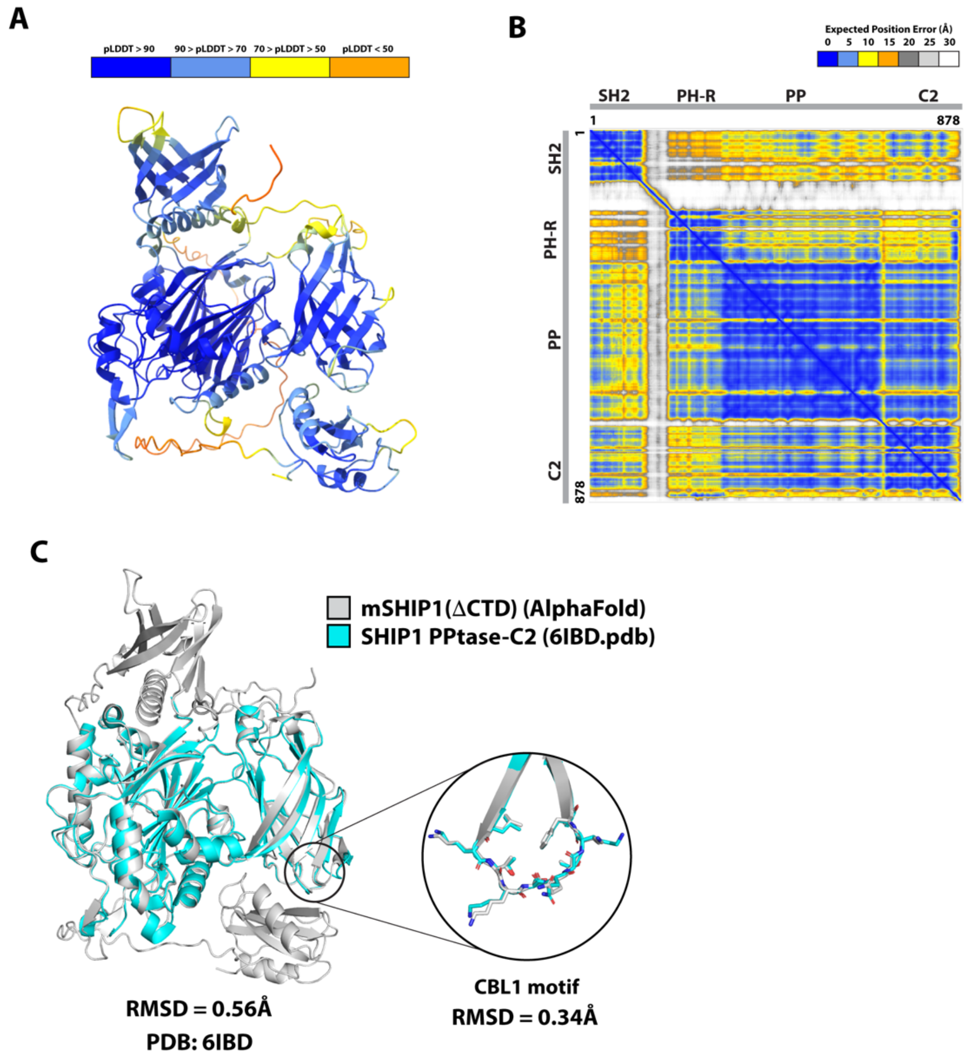

Figure Supplement 3

Generation and validation of an AlphaFold model for monomeric SHIP1(ΔCTD)

(A) AlphaFold2 Multimer prediction of mSHIP1(ΔCTD). Shown below is the model colored by predicted local distance difference (pLDDT) to show regions with high confidence (pLDDT > 90) to low confidence (pLDDT <50). **(B)** Predicted alignment error (PAE) for Alphafold2 multimer model of mini-SHIP1. Note that the PAE plot is not an inter-residue distance map or a contact map. Instead, the coloring indicates expected distance error. **(C)** Overlay of AlphaFold predicted structure of mSHIP1(ΔCTD) (grey) and the X-ray crystal structure human SHIP1 PPtase-C2 (397-857aa, teal). Root mean square deviation (RMSD) for the entire protein and CBL1 motif indicated under the structures.

Supplemental Figure 4

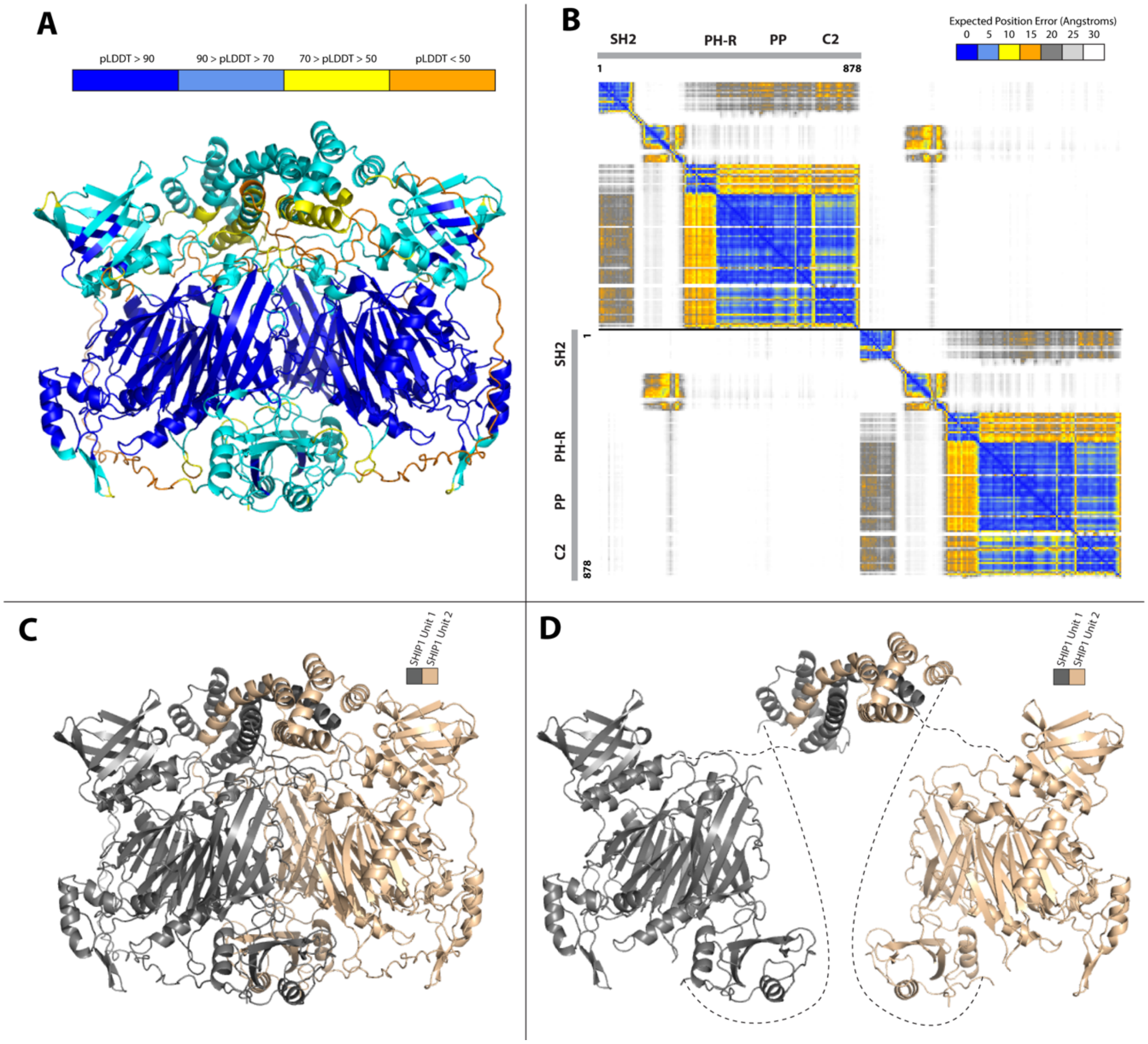

Figure Supplement 4

Generation and validation of an AlphaFold model of the SHIP1(ΔCTD) dimer

(A) AlphaFold 3 search of two copies of SHIP1(ΔCTD). Model is colored by pLDDT to indicate regions of high and low confidence. **(B)** Predicted alignment error (PAE) for AlphaFold3 search of two copies of SHIP1(ΔCTD). The colors indicate the predicted aligned error and are colored according to the legend. **(C)** Structure of SHIP1 dimer colored by chain to differentiate between subunits. Residues with low local confidence (pLDDT < 50) have been removed. **(D)** Modelled interfaces with a high expected position error were manually separated from each other. Low confidence interdomain contacts with the dimerization domain which had been removed from the AlphaFold model were manually annotated using dashed lines.

Supplemental Figure 5

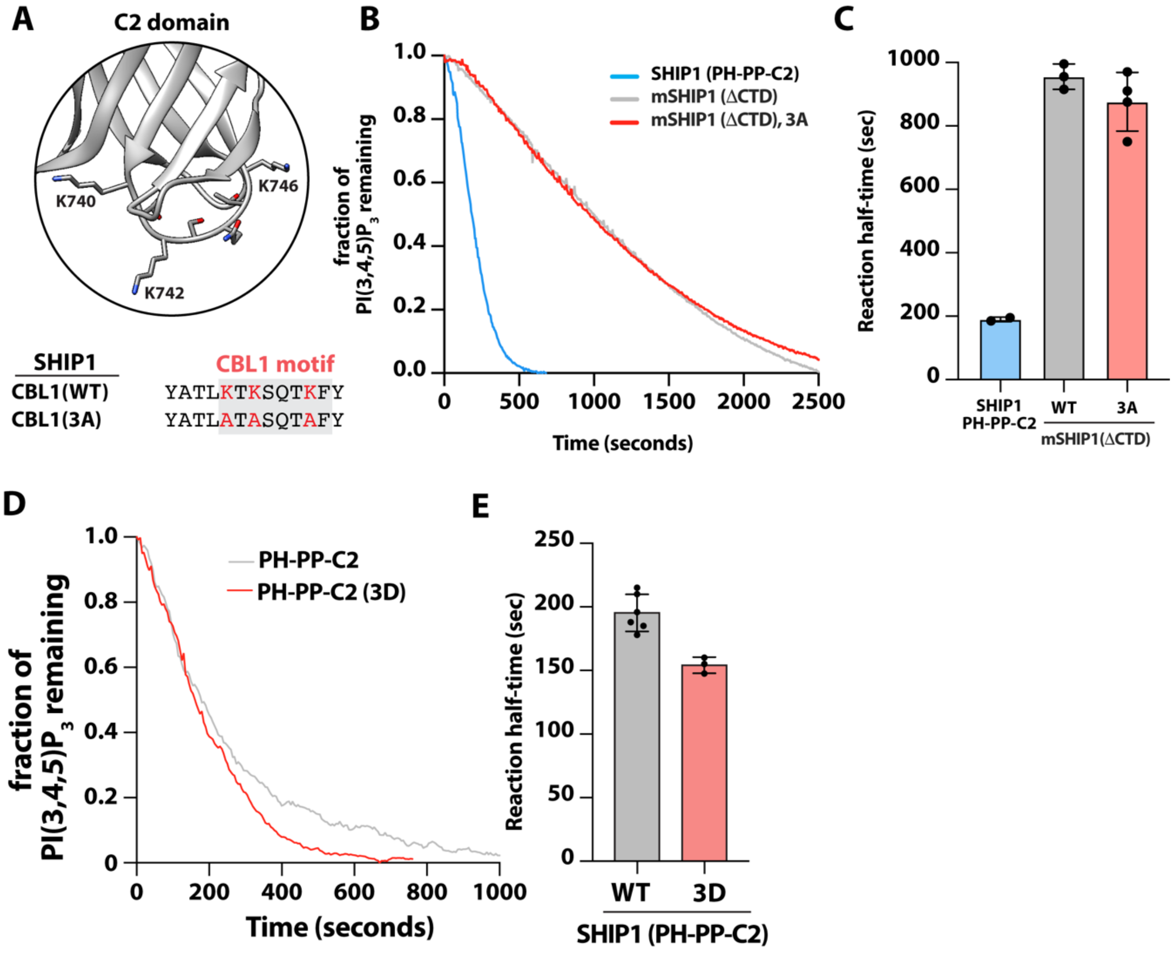

Figure Supplement 5

SHIP1 CBL1 motif mutant lipid phosphatase activity measurements

(A) Structural model of human SHIP1 CBL1 motif with indicated lysine residues (6IBD.pdb). Sequence alignment comparing human SHIP1 CBL1 motif, wild type and mutant (K741A/K743A/K747A, denoted 3A) sequence. **(B)** Charge neutralization (3A) in the CBL1 motif of mini-SHIP1 does not enhance lipid phosphatase activity. Kinetic traces of phosphatase activity measured in the presence of 20 nM mNG-mSHIP1(ΔCTD, WT or 3A) and 20 nM mNG-SHIP1 (PH-PP-C2). **(C)** Quantification of reaction half-times measured in the presence of 20 nM mNG-SHIP1(PH-PP-C2), 20 nM mNG-mSHIP1(ΔCTD), or 20 nM mNG-mSHIP1(ΔCTD, 3A). Bars equal to the mean reaction half-times (N= 3 technical replicates). Errors equal standard deviation. **(D)** Phosphatase measurements of 20 nM mNG-SHIP1(PH-PP-C2) or mNG-SHIP1 CBL1 motif mutants. **(E)** Quantification of reaction half-times of 20 nM mNG-SHIP1(PH-PP-C2) and 20 nM mNG- SHIP1(PH-PP-C2, K741D/K743D/K747D; denoted 3D). Bars are equal to the mean reaction half-times (N = 3-6 technical replicate per construct). Student t-test comparing SHIP1(PH-PP-C2), WT and 3D, produced a p-value = 0.003. (**B-E**) Dephosphorylation of $PI(3,4,5)P_3$ was monitored in the presence of 20 nM AF555-SNAP-Btk. Initial membrane composition: 2% $PI(3,4,5)P_3$, 98% DOPC.

Supplemental Figure 6

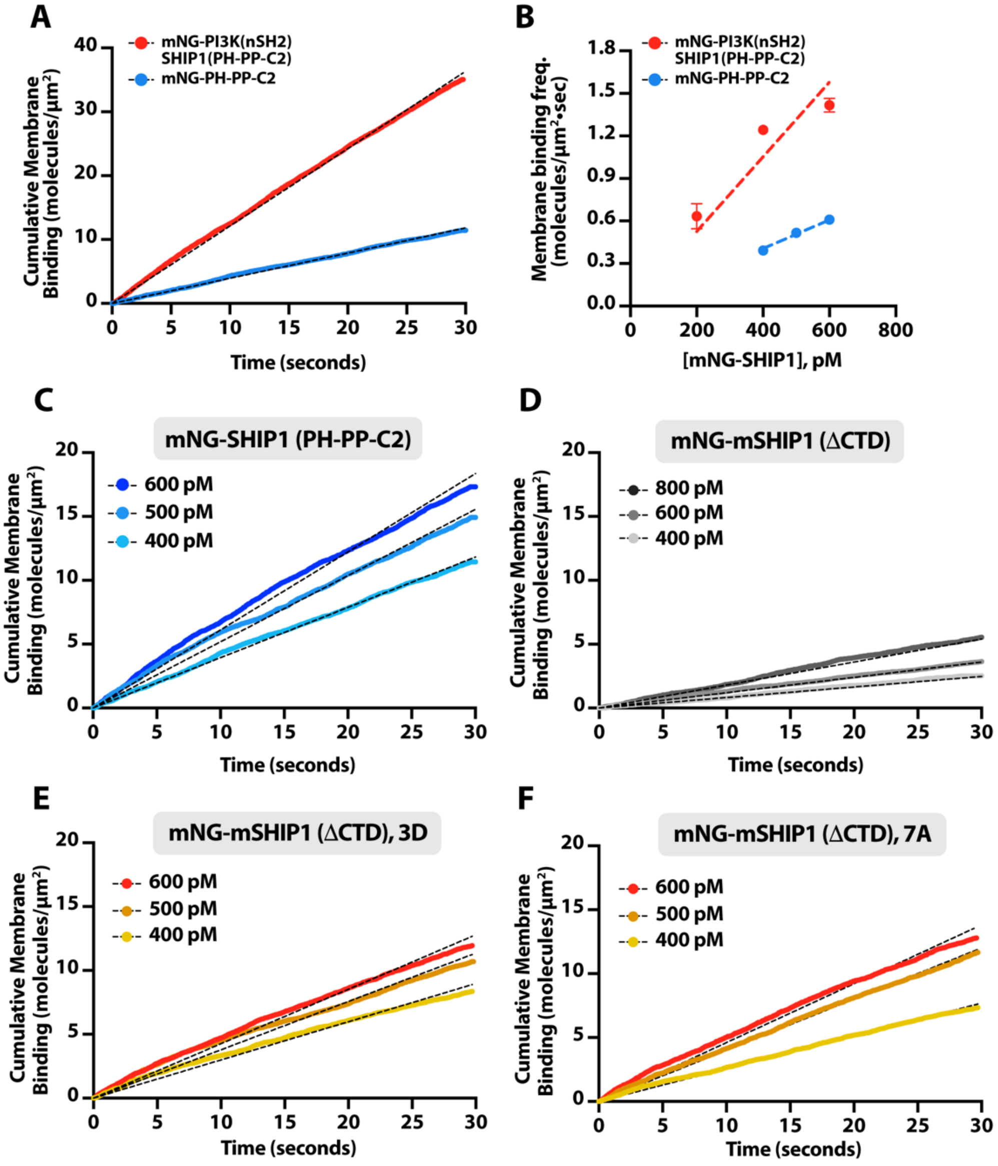

Figure Supplement 6

Single molecule membrane binding frequency measurements

(A) Cumulative binding events measured by TIRF-M in the presence of 400 pM mNG-SHIP1 (PH-PP-C2) and 400 pM mNG-PI3K(SH2)-SHIP1(PH-PP-C2). **(B)** Membrane binding frequency (k_{ON}) calculated from measuring cumulative binding events across the following protein concentrations: 400-600 pM mNG-SHIP1 (PH-PP-C2) and 200-600 pM mNG-PI3K(SH2)-SHIP1(PH-PP-C2). Linear regression yielded the following k_{ON} values: 1.0 nM^{-1}•μm^{-2}•sec^{-1} mNG-SHIP1 (PH-PP-C2) and 2.6 nM^{-1}•μm^{-2}•sec^{-1} mNG-PI3K(SH2)-SHIP1(PH-PP-C2). Cumulative membrane binding events measured in the presence of **(C)** 400-600 pM mNG-SHIP1 (PH-PP-C2), **(D)** 400-800 pM mNG-mSHIP1(ΔCTD), **(E)** 400-600 pM mNG-mSHIP1(ΔCTD, 3D), or **(F)** 400-600 pM mNG-mSHIP1(ΔCTD, 7A). **(A-F)** Membrane composition: 2% PI(3,4,5)P_3, 20% DOPS, 78% DOPC.

Supplemental Figure 7

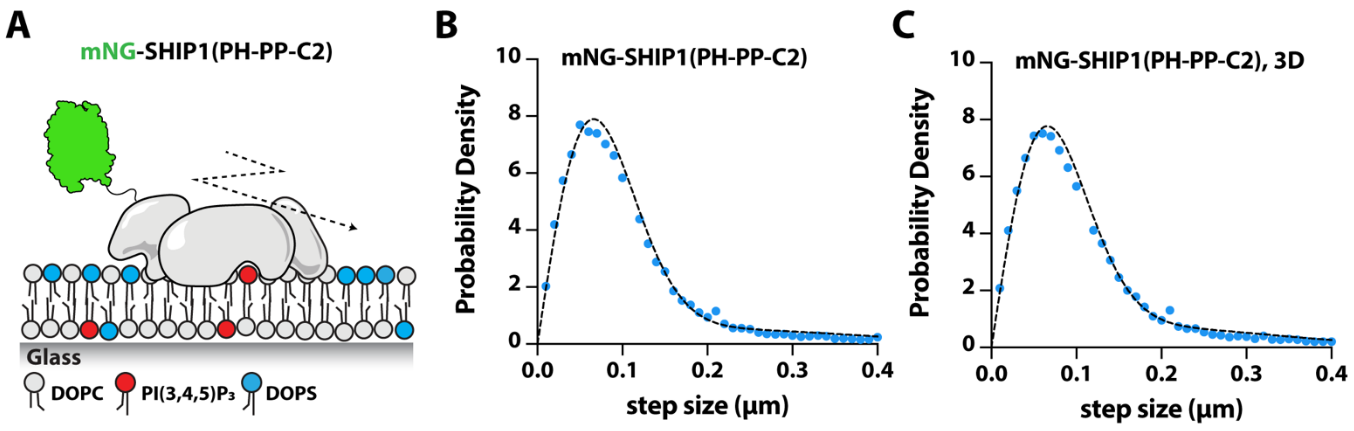

Figure Supplement 7

CBL1 motif mutations do not alter membrane diffusivity of mNG-SHIP1(PH-PP-C2)

(A) Cartoon schematic showing purified mNG-SHIP1(PH-PP-C2) bound to a supported lipid bilayer containing PI(3,4,5)P_3, DOPS, and DOPC. **(B-C)** Step size distributions (or displacement) measured in vitro in the presence of 200 pM mNG-SHIP1(PH-PP-C2) or 200 pM mNG-SHIP1(PH-PP-C2, 3D). The single molecule displacement (µm) was measured between each frame with 12 ms time intervals. **(B-C)** Membrane composition: 2% PI(3,4,5)P_3, 20% DOPS, 78% DOPC.

SUPPLEMENTAL MOVIE LEGENDS

Supplemental Movie 1
Plasma membrane binding dynamics of mEos3.2-SHIP1(PH-PP-C2) (left) and mEos3.2-SHIP1(PH-PP-C2, K741D/K743D/K747D) (right) visualized by smTIRF microscopy in differentiated PLB-985 neutrophil-like cells. Movie is associated with data in Figure 4F-4G. Video plays at 20 frames per second. Data was collected with 22 ms time intervals (i.e. 45 fps). Scale bar is 2 μm.

Supplemental Movie 2
Supported membrane binding dynamics of mNG-mSHIP1(ΔCTD) (left), mNG-SHIP1(ΔCTD) (center), mNG-SHIP1(FL) (right) bound to pY peptides visualized by smTIRF microscopy. Movie is associated with data in Figure 6. Video plays at 10 frames per second. Data was collected with 52 ms time intervals. Scale bar is 2 μm. Membrane composition: 96% DOPC, 2% PI(3,4,5)P_3, 2% MCC-PE(+pY).

Table S1

Protein Data Set	mSHIP1(ΔCTD) Apo	mSHIP1(ΔCTD) Apo + 40 µM pY peptide
HDX reaction details	% D_2O = 66.66%<br>pH(read) = 7.5<br>Temp = 18ºC	% D_2O = 66.66%<br>pH(read) = 7.5<br>Temp = 18ºC
HDX time course	3s, 300s	3s, 300s
HDX controls	N/A	N/A
Back-exchange	Corrected based off % D2O	Corrected based off % D2O
Number of unique peptides	112	112
Sequence coverage	86.3 %	86.3 %
Average peptide length/redundancy	length = 15.2<br>redundancy = 2.23	length = 15.2<br>redundancy = 2.23
Replicates	3	3
Repeatability	Average Stdev = 1.2%	Average Stdev = 1.0%
Significant difference in HDX	> 4.5 % and > 0.45 Da and unpaired t-test < 0.01	> 4.5 % and > 0.45 Da and unpaired t-test < 0.01

Table S1
HDX-MS data and statistics collected for mSHIP1(ΔCTD)
The data analysis statistics for all HDX-MS experiments shown in table were performed and presented according to published guidelines (51). Results are presented as relative levels of deuterium incorporation and the only control for back exchange was the level of deuterium present in the buffer. Differences in exchange in a peptide were considered significant if they met all three of the following criteria: ≥5% change in exchange, ≥0.4 Da difference in exchange, and a p value <0.01 using a two tailed student t-test for mSHIP1(ΔCTD) experiment. The mass spectrometry proteomics data have been deposited to the ProteomeXchange Consortium via the PRIDE partner repository (52) with the dataset PXD061719 for mSHIP1(ΔCTD) data.

Table S2

Protein Data Set	SHIP1 (ΔCTD) Apo	SHIP1 (ΔCTD) Apo + 40 µM pY peptide
HDX reaction details	% D_2O = 70.7%<br>pH(read) = 7.5<br>Temp = 20ºC	% D_2O = 70.7%<br>pH(read) = 7.5<br>Temp = 20ºC
HDX time course	3s, 30s, 300s, 3000s	3s, 30s, 300s, 3000s
HDX controls	N/A	N/A
Back-exchange	Corrected based off % D2O	Corrected based off % D2O
Number of unique peptides	156	156
Sequence coverage	86 %	86 %
Average peptide length/redundancy	length = 15.5<br>redundancy = 2.1	length = 15.5<br>redundancy = 2.1
Replicates	3	3
Repeatability	Average Stdev = 1.1%	Average Stdev = 0.9%
Significant difference in HDX	> 5 % and > 0.4 Da and unpaired t-test < 0.01	> 5 % and > 0.4 Da and unpaired t-test < 0.01

Table S2
HDX-MS data and statistics collected for SHIP1(1-878aa, ΔCTD)
The data analysis statistics for all HDX-MS experiments shown in table were performed and presented according to published guidelines (51). Results are presented as relative levels of deuterium incorporation and the only control for back exchange was the level of deuterium present in the buffer. Differences in exchange in a peptide were considered significant if they met all three of the following criteria: ≥5% change in exchange, ≥0.4 Da difference in exchange, and a p value <0.01 using a two tailed student t-test for SHIP1(ΔCTD) experiment. The mass spectrometry proteomics data have been deposited to the ProteomeXchange Consortium via the PRIDE partner repository (52) with the dataset identifier PXD058704 for SHIP1(ΔCTD) data.

PLASMID INVENTORY

Recombinant DNA			
his6-SUMO-Btk(PH-TH,R49S/K52S)-SNAP	bacterial	Duewell et al. 2024	pSH1313
his10-TEV-**mNG**-GGGGG-SHIP1 (PH-PP-C2, 292-878aa)	bacterial	Waddell et al. 2023	pSH1082
his10-TEV-**mNG**-GGGGG-SHIP1 (PH-PP-C2, 292-878aa) (K741D, K743D, K747D)	bacterial	This paper	pSH1405
his10-TEV-**mNG**-GGGGG-SHIP1 (Rho binding domain,152-291aa)	Bacterial	This paper	pSH1295
his10-TEV-**mNG**	bacterial	This paper	pSH917
his6-TEV-**mNG**-SHIP1 FL (1-1189aa)	baculovirus	Waddell et al. 2023	pSH973
his6-TEV-**mNG**-SHIP1 ΔCTD (1-878aa)	baculovirus	Waddell et al. 2023	pSH1042
“mSHIP1 ΔCTD” = SHIP1 SH2-PH-PP-C2 (1-158aa, 292-878aa)			
his6-TEV-**mNG**-mSHIP1 ΔCTD	baculovirus	This paper	pSH1327
his6-TEV-**mNG**-mSHIP1 ΔCTD (3A = K741A, K743A, K747A)	baculovirus	This paper	pSH1369
his6-TEV-**mNG**-mSHIP1 ΔCTD (3D = K741D, K743D, K747D)	Baculovirus	This paper	pSH1390
his6-TEV-**mNG**-mSHIP1 ΔCTD (7A = K741A, T742A, K743A, S744A, Q745A, T746A, K747A)	Baculovirus	This paper	pSH1397
his6-TEV-**mNG**-mSHIP1 ΔCTD (PIK3R1 SH2 swap) (PIK3R1 322-440aa)-(SHIP1 102-158aa)-(SHIP1 PH-PP-C2 292-878aa)	Baculovirus	This paper	pSH1414
psPAX2 (2nd generation lentiviral packaging vector)	lentivirus	Addgene, 12260	pSH1224
pVSV-G (VSV-G envelop protein)	lentivirus	Addgene, 138479	pSH1226
Ubc-**mEos3.2**-(GGGGS)x2-SHIP1 (PH-PP-C2, 292-878aa)	lentivirus	Waddell et al. 2023	pSH1254
Ubc-**mEos3.2**-(GGGGS)x2-SHIP1 (PH-PP-C2) (K741D, K743D, K747D	lentivirus	This paper	pSH1447

PEPTIDE SEQUENCES

The protein sequences of recombinantly expressed and purified proteins used in the study are shown below. The following tags were cleaved off the recombinant proteins and removed during the purification:

his6-TEV-SUMO (↓= site of SUMO protease (Ulp1) cleavage)
MGSSHHHHHHSSGLVPRGSHMASMSDSEVNQEAKPEVKPEVKPETHINLKVSDGSSEIFFKIKKTTPL
RRLMEAFAKRQGKEMDSLRFLYDGIRIQADQTPEDLDMEDNDIIEAHREQIGG↓SEF

his6-TEV (↓= site of TEV protease cleavage)
MGSSHHHHHHENLYFQ↓SN

Shown below are the plasmid #'s and names of the full-length recombinant proteins that were expressed and purified in this study. The affinity and solubility tags that are underlined in the gene names were cleaved off the indicated protein and removed through purification. The primary amino acid sequence shown below represents the final purified product.

pSH1313, his6-SUMO-Btk(PH-TH,R49S/K52S)-SNAP
…SEFMATVILESIFLKRSQQKKKTSPLNFKKRLFLLTVQKLSYYEYDFERGRSGSSKGSIDVEKITCVET
VVPEKNPPPERQIPRRGEESSETEQISIIERFPYPFQVVYDEGPLYVFSPTEELRKRWIHQLKNVIRYNS
DLVQKYHPCFWIDGQYLCCSQTAKNAMGCQILENRSGRGDKDCEMKRTTLDSPLGKLELSGCEQGLH
EIKLLGKGTSAADAVEVPAPAAVLGGPEPLMQATAWLNAYFHQPEAIEEFPVPALHHPVFQQESFTRQ
VLWKLLKVVKFGEVISYQQLAALAGNPAATAAVKTALSGNPVPILIPCHRVVSSSGAVGGYEGGLAVKE
WLLAHEGHRLGKPGLG*

pSH973, his6-TEV-mNeonGreen-(GGGGS)x2-SHIP1 FL (1-1189aa)
…SNTGMVSKGEEDNMASLPATHELHIFGSINGVDFDMVGQGTGNPNDGYEELNLKSTKGDLQFSPWI
LVPHIGYGFHQYLPYPDGMSPFQAAMVDGSGYQVHRTMQFEDGASLTVNYRYTYEGSHIKGEAQVK
GTGFPADGPVMTNSLTAADWCRSKKTYPNDKTIISTFKWSYTTGNGKRYRSTARTTYTFAKPMAANYL
KNQPMYVFRKTELKHSKTELNFKEWQKAFTDVMGMDELYKGGGGSGGGGSTSVPCWNHGNITRSK
AEELLSRTGKDGSFLVRASESISRAYALCVLYRNCVYTYRILPNEDDKFTVQASEGVSMRFFTKLDQLIE
FYKKENMGLVTHLQYPVPLEEEDTGDDPEEDTESVVSPPELPPRNIPLTASSCEAKEVPFSNENPRAT
ETSRPSLSETLFQRLQSMDTSGLPEEHLKAIQDYLSTQLAQDSEFVKTGSSSLPHLKKLTTLLCKELYG
EVIRTLPSLESLQRLFDQQLSPGLRPRPQVPGEANPINMVSKLSQLTSLLSSIEDKVKALLHEGPESPHR
PSLIPPVTFEVKAESLGIPQKMQLKVDVESGKLIIKKSKDGSEDKFYSHKKILQLIKSQKFLNKLVILVETE
KEKILRKEYVFADSKKREGFCQLLQQMKNKHSEQPEPDMITIFIGTWNMGNAPPPKKITSWFLSKGQG
KTRDDSADYIPHDIYVIGTQEDPLSEKEWLEILKHSLQEITSVTFKTVAIHTLWNIRIVVLAKPEHENRISHI
CTDNVKTGIANTLGNKGAVGVSFMFNGTSLGFVNSHLTSGSEKKLRRNQNYMNILRFLALGDKKLSPF
NITHRFTHLFWFGDLNYRVDLPTWEAETIIQKIKQQQYADLLSHDQLLTERREQKVFLHFEEEEITFAPT
YRFERLTRDKYAYTKQKATGMKYNLPSWCDRVLWKSYPLVHVVCQSYGSTSDIMTSDHSPVFATFEA
GVTSQFVSKNGPGTVDSQGQIEFLRCYATLKTKSQTKFYLEFHSSCLESFVKSQEGENEEGSEGELVV
KFGETLPKLKPIISDPEYLLDQHILISIKSSDSDESYGEGCIALRLEATETQLPIYTPLTHHGELTGHFQGEI
KLQTSQGKTREKLYDFVKTERDESSGPKTLKSLTSHDPMKQWEVTSRAPPCSGSSITEIINPNYMGVG
PFGPPMPLHVKQTLSPDQQPTAWSYDQPPKDSPLGPCRGESPPTPPGQPPISPKKFLPSTANRGLPP
RTQESRPSDLGKNAGDTLPQEDLPLTKPEMFENPLYGSLSSFPKPAPRKDQESPKMPRKEPPPCPEP
GILSPSIVLTKAQEADRGEGPGKQVPAPRLRSFTCSSSAEGRAAGGDKSQGKPKTPVSSQAPVPAKRP
IKPSRSEINQQTPPTPTPRPPLPVKSPAVLHLQHSKGRDYRDNTELPHHGKHRPEEGPPGPLGRTAMQ
*

pSH1042, his6-TEV-mNeonGreen-(GGGGS)x2-SHIP1 (ΔCTD) (1-878aa)
…SNTGMVSKGEEDNMASLPATHELHIFGSINGVDFDMVGQGTGNPNDGYEELNLKSTKGDLQFSPWI
LVPHIGYGFHQYLPYPDGMSPFQAAMVDGSGYQVHRTMQFEDGASLTVNYRYTYEGSHIKGEAQVK

GTGFPADGPVMTNSLTAADWCRSKKTYPNDKTIISTFKWSYTTGNGKRYRSTARTTYTFAKPMAANYL
KNQPMYVFRKTELKHSKTELNFKEWQKAFTDVMGMDELYKGGGGSGGGGSTSVPCWNHGNITRSK
AEELLSRTGKDGSFLVRASESISRAYALCVLYRNCVYTYRILPNEDDKFTVQASEGVSMRFFTKLDQLIE
FYKKENMGLVTHLQYPVPLEEEDTGDDPEEDTESVVSPPELPPRNIPLTASSCEAKEVPFSNENPRAT
ETSRPSLSETLFQRLQSMDTSGLPEEHLKAIQDYLSTQLAQDSEFVKTGSSSLPHLKKLTTLLCKELYG
EVIRTLPSLESLQRLFDQQLSPGLRPRPQVPGEANPINMVSKLSQLTSLLSSIEDKVKALLHEGPESPHR
PSLIPPVTFEVKAESLGIPQKMQLKVDVESGKLIIKKSKDGSEDKFYSHKKILQLIKSQKFLNKLVILVETE
KEKILRKEYVFADSKKREGFCQLLQQMKNKHSEQPEPDMITIFIGTWNMGNAPPPKKITSWFLSKGQG
KTRDDSADYIPHDIYVIGTQEDPLSEKEWLEILKHSLQEITSVTFKTVAIHTLWNIRIVVLAKPEHENRISHI
CTDNVKTGIANTLGNKGAVGVSFMFNGTSLGFVNSHLTSGSEKKLRRNQNYMNILRFLALGDKKLSPF
NITHRFTHLFWFGDLNYRVDLPTWEAETIIQKIKQQQYADLLSHDQLLTERREQKVFLHFEEEEITFAPT
YRFERLTRDKYAYTKQKATGMKYNLPSWCDRVLWKSYPLVHVVCQSYGSTSDIMTSDHSPVFATFEA
GVTSQFVSKNGPGTVDSQGQIEFLRCYATLKTKSQTKFYLEFHSSCLESFVKSQEGENEEGSEGELVV
KFGETLPKLKPIISDPEYLLDQHILISIKSSDSDESYGEGCIALRLEATETQLPIYTPLTHHGELTGHFQGEI
KLQTSQGKTREKLYDFVKTERDESSGPK*

pSH1082, <u>his10-TEV</u>-mNeonGreen-(GGGGG)-SHIP1 PH-PP-C2 (292-878aa)
…GAMVSKGEEDNMASLPATHELHIFGSINGVDFDMVGQGTGNPNDGYEELNLKSTKGDLQFSPWILV
PHIGYGFHQYLPYPDGMSPFQAAMVDGSGYQVHRTMQFEDGASLTVNYRYTYEGSHIKGEAQVKGT
GFPADGPVMTNSLTAADWCRSKKTYPNDKTIISTFKWSYTTGNGKRYRSTARTTYTFAKPMAANYLKN
QPMYVFRKTELKHSKTELNFKEWQKAFTDVMGMDELYKGGGGGTSPSLIPPVTFEVKAESLGIPQKM
QLKVDVESGKLIIKKSKDGSEDKFYSHKKILQLIKSQKFLNKLVILVETEKEKILRKEYVFADSKKREGFCQ
LLQQMKNKHSEQPEPDMITIFIGTWNMGNAPPPKKITSWFLSKGQGKTRDDSADYIPHDIYVIGTQEDP
LSEKEWLEILKHSLQEITSVTFKTVAIHTLWNIRIVVLAKPEHENRISHICTDNVKTGIANTLGNKGAVGVS
FMFNGTSLGFVNSHLTSGSEKKLRRNQNYMNILRFLALGDKKLSPFNITHRFTHLFWFGDLNYRVDLPT
WEAETIIQKIKQQQYADLLSHDQLLTERREQKVFLHFEEEEITFAPTYRFERLTRDKYAYTKQKATGMKY
NLPSWCDRVLWKSYPLVHVVCQSYGSTSDIMTSDHSPVFATFEAGVTSQFVSKNGPGTVDSQGQIEF
LRCYATLKTKSQTKFYLEFHSSCLESFVKSQEGENEEGSEGELVVKFGETLPKLKPIISDPEYLLDQHILI
SIKSSDSDESYGEGCIALRLEATETQLPIYTPLTHHGELTGHFQGEIKLQTSQGKTREKLYDFVKTERDE
SSGPK*

pSH1327, <u>his6-TEV</u>-mNeonGreen-(GGGGS)$_{x2}$-mSHIP1(ΔCTD, 1-158aa + 292-878aa)
…SNTGMVSKGEEDNMASLPATHELHIFGSINGVDFDMVGQGTGNPNDGYEELNLKSTKGDLQFSPWI
LVPHIGYGFHQYLPYPDGMSPFQAAMVDGSGYQVHRTMQFEDGASLTVNYRYTYEGSHIKGEAQVK
GTGFPADGPVMTNSLTAADWCRSKKTYPNDKTIISTFKWSYTTGNGKRYRSTARTTYTFAKPMAANYL
KNQPMYVFRKTELKHSKTELNFKEWQKAFTDVMGMDELYKGGGGSGGGGSTSVPCWNHGNITRSK
AEELLSRTGKDGSFLVRASESISRAYALCVLYRNCVYTYRILPNEDDKFTVQASEGVSMRFFTKLDQLIE
FYKKENMGLVTHLQYPVPLEEEDTGDDPEEDTESVVSPPELPPRNIPLTASSCEAKEVPFSNENPRAT
ETSRPSTSPSLIPPVTFEVKAESLGIPQKMQLKVDVESGKLIIKKSKDGSEDKFYSHKKILQLIKSQKFLN
KLVILVETEKEKILRKEYVFADSKKREGFCQLLQQMKNKHSEQPEPDMITIFIGTWNMGNAPPPKKITSW
FLSKGQGKTRDDSADYIPHDIYVIGTQEDPLSEKEWLEILKHSLQEITSVTFKTVAIHTLWNIRIVVLAKPE
HENRISHICTDNVKTGIANTLGNKGAVGVSFMFNGTSLGFVNSHLTSGSEKKLRRNQNYMNILRFLALG
DKKLSPFNITHRFTHLFWFGDLNYRVDLPTWEAETIIQKIKQQQYADLLSHDQLLTERREQKVFLHFEEE
EITFAPTYRFERLTRDKYAYTKQKATGMKYNLPSWCDRVLWKSYPLVHVVCQSYGSTSDIMTSDHSPV
FATFEAGVTSQFVSKNGPGTVDSQGQIEFLRCYATLKTKSQTKFYLEFHSSCLESFVKSQEGENEEGS
EGELVVKFGETLPKLKPIISDPEYLLDQHILISIKSSDSDESYGEGCIALRLEATETQLPIYTPLTHHGELTG
HFQGEIKLQTSQGKTREKLYDFVKTERDESSGPK*

pSH1414 <u>his6-TEV</u>-mNeonGreen-(GGGGS)$_{x2}$- mSHIP1(ΔCTD, PIK3R1 SH2 domain swap) (PIK3R1 322-440aa)-(SHIP1 102-158aa)-(SHIP1 PH-PP-C2 292-878aa)
…SNTGMVSKGEEDNMASLPATHELHIFGSINGVDFDMVGQGTGNPNDGYEELNLKSTKGDLQFSPWI
LVPHIGYGFHQYLPYPDGMSPFQAAMVDGSGYQVHRTMQFEDGASLTVNYRYTYEGSHIKGEAQVK
GTGFPADGPVMTNSLTAADWCRSKKTYPNDKTIISTFKWSYTTGNGKRYRSTARTTYTFAKPMAANYL
KNQPMYVFRKTELKHSKTELNFKEWQKAFTDVMGMDELYKGGGGSGGGGSTSNNNMSLQDAEWY

WGDISREEVNEKLRDTADGTFLVRDASTKMHGDYTLTLRKGGNNKLIKIFHRDGKYGFSDPLTFSSVVE
LINHYRNESLAQYNPKLDVKLLYPVSKYQQDQVVKEDPLEEEDTGDDPEEDTESVVSPPELPPRNIPLT
ASSCEAKEVPFSNENPRATETSRPSTSPSLIPPVTFEVKAESLGIPQKMQLKVDVESGKLIIKKSKDGSE
DKFYSHKKILQLIKSQKFLNKLVILVETEKEKILRKEYVFADSKKREGFCQLLQQMKNKHSEQPEPDMITI
FIGTWNMGNAPPPKKITSWFLSKGQGKTRDDSADYIPHDIYVIGTQEDPLSEKEWLEILKHSLQEITSVT
FKTVAIHTLWNIRIVVLAKPEHENRISHICTDNVKTGIANTLGNKGAVGVSFMFNGTSLGFVNSHLTSGS
EKKLRRNQNYMNILRFLALGDKKLSPFNITHRFTHLFWFGDLNYRVDLPTWEAETIIQKIKQQQYADLLS
HDQLLTERREQKVFLHFEEEEITFAPTYRFERLTRDKYAYTKQKATGMKYNLPSWCDRVLWKSYPLVH
VVCQSYGSTSDIMTSDHSPVFATFEAGVTSQFVSKNGPGTVDSQGQIEFLRCYATLKTKSQTKFYLEF
HSSCLESFVKSQEGENEEGSEGELVVKFGETLPKLKPIISDPEYLLDQHILISIKSSDSDESYGEGCIALR
LEATETQLPIYTPLTHHGELTGHFQGEIKLQTSQGKTREKLYDFVKTERDESSGPK*

pSH1295 his10-TEV-mNeonGreen-(GGGGG)-SHIP1 Rho-binding domain (152-291aa)
…GAMVSKGEEDNMASLPATHELHIFGSINGVDFDMVGQGTGNPNDGYEELNLKSTKGDLQFSPWILV
PHIGYGFHQYLPYPDGMSPFQAAMVDGSGYQVHRTMQFEDGASLTVNYRYTYEGSHIKGEAQVKGT
GFPADGPVMTNSLTAADWCRSKKTYPNDKTIISTFKWSYTTGNGKRYRSTARTTYTFAKPMAANYLKN
QPMYVFRKTELKHSKTELNFKEWQKAFTDVMGMDELYKGGGGGTSATETSRPSLSETLFQRLQSMD
TSGLPEEHLKAIQDYLSTQLAQDSEFVKTGSSSLPHLKKLTTLLCKELYGEVIRTLPSLESLQRLFDQQL
SPGLRPRPQVPGEANPINMVSKLSQLTSLLSSIEDKVKALLHEGPESPH*

pSH1254, mEos3.2-(GGGGGS)x2-SHIP1 PH-PP-C2 (292-878aa), lentiviral vector
MSAIKPDMKIKLRMEGNVNGHHFVIDGDGTGKPFEGKQSMDLKVKEGGPLPFAFDILTTAFHYGNRVF
AKYPDNIQDYFKQSFPKGYSWERSLTFEDGGICNARNDITMEGDTFYNKVRFYGTNFPANGPVMQKK
TLKWEPSTEKMYVRDGVLTGDIEMALLLEGNAHYRCDFRTTYKAKEKGVKLPGAHFVDHCIEILSHDKD
YNKVKLYEHAVAHSGLPDNARRGGGGSGGGGSTGPSLIPPVTFEVKAESLGIPQKMQLKVDVESGKLII
KKSKDGSEDKFYSHKKILQLIKSQKFLNKLVILVETEKEKILRKEYVFADSKKREGFCQLLQQMKNKHSE
QPEPDMITIFIGTWNMGNAPPPKKITSWFLSKGQGKTRDDSADYIPHDIYVIGTQEDPLSEKEWLEILKH
SLQEITSVTFKTVAIHTLWNIRIVVLAKPEHENRISHICTDNVKTGIANTLGNKGAVGVSFMFNGTSLGFV
NSHLTSGSEKKLRRNQNYMNILRFLALGDKKLSPFNITHRFTHLFWFGDLNYRVDLPTWEAETIIQKIKQ
QQYADLLSHDQLLTERREQKVFLHFEEEEITFAPTYRFERLTRDKYAYTKQKATGMKYNLPSWCDRVL
WKSYPLVHVVCQSYGSTSDIMTSDHSPVFATFEAGVTSQFVSKNGPGTVDSQGQIEFLRCYATLKTKS
QTKFYLEFHSSCLESFVKSQEGENEEGSEGELVVKFGETLPKLKPIISDPEYLLDQHILISIKSSDSDESY
GEGCIALRLEATETQLPIYTPLTHHGELTGHFQGEIKLQTSQGKTREKLYDFVKTERDESSGPK*

pSH1267, mEos3.2-(GGGGGS)x2-LactC2, lentiviral vector
MSAIKPDMKIKLRMEGNVNGHHFVIDGDGTGKPFEGKQSMDLKVKEGGPLPFAFDILTTAFHYGNRVF
AKYPDNIQDYFKQSFPKGYSWERSLTFEDGGICNARNDITMEGDTFYNKVRFYGTNFPANGPVMQKK
TLKWEPSTEKMYVRDGVLTGDIEMALLLEGNAHYRCDFRTTYKAKEKGVKLPGAHFVDHCIEILSHDKD
YNKVKLYEHAVAHSGLPDNARRGGGGSGGGGSTGCTEPLGLKDNTIPNKQITASSYYKTWGLSAFSW
FPYYARLDNQGKFNAWTAQTNSASEWLQIDLGSQKRVTGIITQGARDFGHIQYVAAYRVAYGDDGVT
WTEYKDPGASESKIFPGNMDNNSHKKNIFETPFQARFVRIQPVAWHNRITLRVELLGC*
